# Distinctive exercise-induced inflammatory response and exerkine induction in skeletal muscle of people with type 2 diabetes

**DOI:** 10.1126/sciadv.abo3192

**Published:** 2022-09-07

**Authors:** Nicolas J. Pillon, Jonathon A. B. Smith, Petter S. Alm, Alexander V. Chibalin, Julia Alhusen, Erik Arner, Piero Carninci, Tomas Fritz, Julia Otten, Tommy Olsson, Sophie van Doorslaer de ten Ryen, Louise Deldicque, Kenneth Caidahl, Harriet Wallberg-Henriksson, Anna Krook, Juleen R. Zierath

**Affiliations:** ^1^ Department of Physiology and Pharmacology, Karolinska Institutet, 171 77 Stockholm, Sweden.; ^2^ Department of Molecular Medicine and Surgery, Karolinska Institutet, 171 76 Stockholm, Sweden.; ^3^ RIKEN Center for Integrative Medical Sciences, Yokohama, Kanagawa 230-0045, Japan.; ^4^ Centre for Family and Community Medicine, Karolinska Institutet, Huddinge, Sweden.; ^5^ Department of Public Health and Clinical Medicine, Umeå University, Umeå, Sweden.; ^6^ Institute of Neuroscience, UCLouvain, Louvain-la-Neuve, Belgium.

## Abstract

Mechanistic insights into the molecular events by which exercise enhances the skeletal muscle phenotype are lacking, particularly in the context of type 2 diabetes. Here, we unravel a fundamental role for exercise-responsive cytokines (*exerkines*) on skeletal muscle development and growth in individuals with normal glucose tolerance or type 2 diabetes. Acute exercise triggered an inflammatory response in skeletal muscle, concomitant with an infiltration of immune cells. These exercise effects were potentiated in type 2 diabetes. In response to contraction or hypoxia, cytokines were mainly produced by endothelial cells and macrophages. The chemokine CXCL12 was induced by hypoxia in endothelial cells, as well as by conditioned medium from contracted myotubes in macrophages. We found that CXCL12 was associated with skeletal muscle remodeling after exercise and differentiation of cultured muscle. Collectively, acute aerobic exercise mounts a noncanonical inflammatory response, with an atypical production of exerkines, which is potentiated in type 2 diabetes.

## Teaser

Exercise triggers a noncanonical inflammatory response in people with type 2 diabetes, with an atypical production of exerkines.

## INTRODUCTION

Type 2 diabetes is characterized by insulin resistance and altered mitochondrial energetics, concomitant with an aging-related loss of skeletal muscle mass that is exacerbated by low levels of physical activity (*1*). People with type 2 diabetes are often less physically fit than age- and weight-matched normal glucose-tolerant counterparts (*2*). The extent to which this is due to reduced habitual exercise or an attenuated response to exercise training is unclear. Nevertheless, physical exercise and diet manipulation, interventions for type 2 diabetes with high preventative and therapeutic value, have little or no adverse side effects (*3*). Exercise training improves whole-body energy homeostasis by enhancing skeletal muscle metabolic flexibility and insulin sensitivity and is therefore an efficacious intervention to improve glucose regulation in individuals with type 2 diabetes (*3*). Aerobic training has traditionally been the predominant mode of exercise prescribed for the clinical management of type 2 diabetes (*4*), but high-intensity interval training and resistance training regimes are also effective to increase skeletal muscle insulin sensitivity and whole-body blood glucose control (*5*, *6*).

Improved fitness results from the repetition of acute bouts of exercise over long periods of time. Each exercise bout is an acute mechanical, hormonal, and metabolic stress that activates signaling pathways in multiple organs (*7*). Tissues involved in whole-body metabolic homeostasis respond to acute exercise by switching from anabolism to catabolism: Lipolysis is activated in adipose tissue, while skeletal muscle uses glycogen, in addition to fatty acids and glucose from the circulation (*8*). Apart from the metabolic perturbations, acute exercise activates transcription in skeletal muscle (*9*), but mechanistic insights into the specific molecular and cellular events by which exercise training enhances insulin sensitivity and preserves muscle mass are lacking, particularly in the context of type 2 diabetes.

We hypothesized that transcriptomic changes in skeletal muscle would be qualitatively and/or quantitatively altered by the metabolic status of the participant and that exercise would therefore induce different pathways in people with normal glucose tolerance (NGT) versus type 2 diabetes. Using transcriptomic and biochemical assays in human skeletal muscle and cell culture models, we unraveled a fundamental role for noncanonical exercise-responsive inflammatory responses in individuals with type 2 diabetes. We provide evidence for a distinctive cytokine induction in skeletal muscle of men with type 2 diabetes and highlight CXCL12 as an exerkine promoting skeletal muscle development and growth.

## RESULTS

### Exercise triggers waves of transcription associated with inflammatory and metabolic processes

**Fig. 1.**
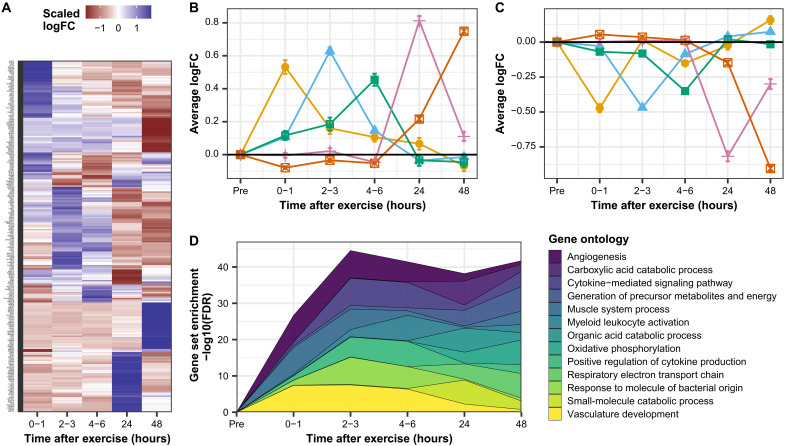
Exercise triggers waves of transcription associated with inflammation and metabolic processes. (**A**) Gene expression changes in skeletal muscle after exercise were collected from the MetaMEx database and processed as described in Materials and Methods. Heatmap of the logFC of genes regulated at each time point. The top 100 genes ranked on FDR are presented. (**B** and **C**) Peak time for each significant gene was calculated and the log(fold change) of genes with the same peak time averaged. Orange, average of genes peaking 0 to 1 hour after exercise; blue, average of genes peaking 2 to 3 hours after exercise; green, average of genes peaking 4 to 6 hours after exercise; pink, average of genes peaking 24 hours after exercise; and red, average of genes peaking 48 hours after exercise. (**D**) Gene set enrichment analysis was performed on logFC-ranked genes at each time point. The top 3 gene ontology terms based on FDR for each time point are presented.

We generated a transcriptional timeline from 0 to 48 hours after acute exercise in skeletal muscle of healthy individuals using the MetaMEx database (*9*). Each time point after exercise was associated with a defined transcriptomic profile (Fig. 1A). Most exercise-responsive genes [false discovery rate (FDR) < 0.1] exhibited transient changes, with a defined peak time and restoration to baseline after a few hours (Fig. 1, B and C). Gene ontology pathways associated with transcripts activated at each time point showed that blood vessel activation and skeletal muscle system processes were induced first (Fig. 1D). Genes associated with inflammatory response and cytokine signaling were induced 2 to 3 hours after exercise, and these profiles were maintained throughout 4 to 6 hours. At 24 and 48 hours after exercise, most genes were associated with metabolic processes, such as oxidative phosphorylation (Fig. 1D). Our meta-analysis reveals how exercise induces waves of transcription and provides evidence to suggest that activation of inflammatory pathways and infiltration of skeletal muscle by immune cells occur rapidly after a single bout of exercise in healthy individuals.

### Discrete skeletal muscle transcriptomic response to exercise in men with type 2 diabetes

To test whether the metabolic status of an individual ablates or synergizes with exercise-induced inflammation, 20 men with type 2 diabetes and 17 men with NGT were recruited, matched for age, weight, and percentage body fat. A first biopsy was taken from the participants in the resting state. Thereafter, the participants performed a 30-min bout of cycling at 85% of their previously determined maximal heart rate, and skeletal muscle biopsies were collected immediately after (post) and 3 hours after exercise (recovery). The transcriptome of skeletal muscle was analyzed by RNA sequencing.

**Fig. 2.**
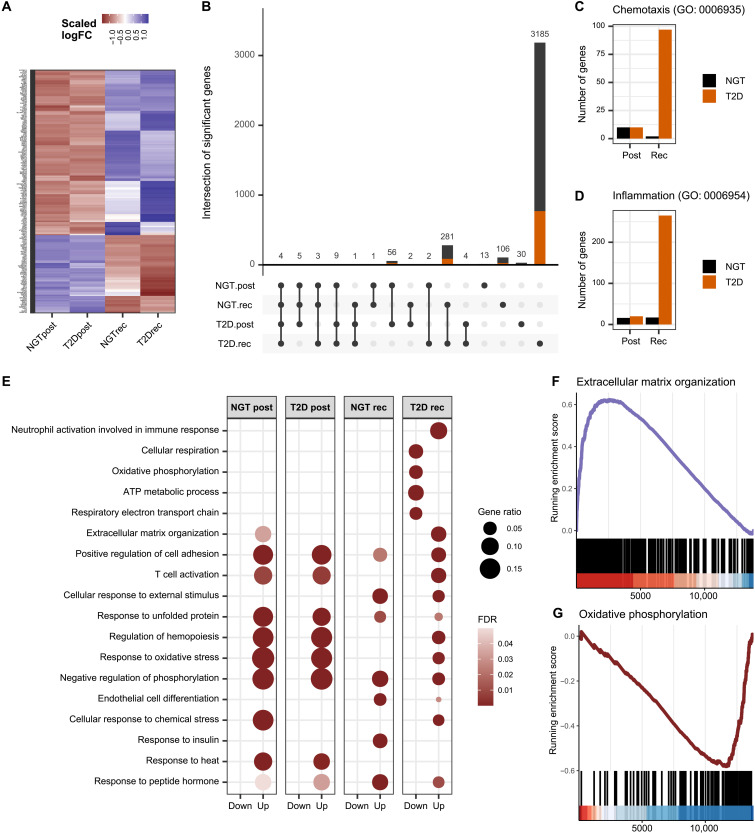
Enhanced inflammatory response to exercise in skeletal muscle from men with type 2 diabetes. (**A**) Heatmap of the logFC of genes significantly regulated by exercise in either normal glucose tolerance (NGT; *n* = 17) or type 2 diabetes (T2D; *n* = 20). (**B**) Number of genes regulated by exercise (FDR < 0.01) and their intersection. Orange, genes annotated with inflammatory processes in gene ontology. (**C** and **D**) Number of genes associated with the gene ontology pathways “chemotaxis” and “inflammatory response” increased after exercise (FDR < 0.01). (**E**) Exercise-responsive gene ontology biological processes calculated with an overrepresentation test. (**F** and **G**) Gene set enrichment analysis performed on genes ranked based on log(fold change) interaction effect exercise*T2D at the recovery time point.

Consistent with the timeline analysis (Fig. 1), exercise-induced changes in gene expression in skeletal muscle differed between the post and recovery time points (Fig. 2A). Exercise triggered a marked increase in skeletal muscle gene expression after recovery in men with type 2 diabetes, with 3185 genes unique to this group (Fig. 2B). While both diagnosis groups showed an overall similar response post exercise, the transcriptional response of skeletal muscle in men with type 2 diabetes was quantitatively larger during recovery. Many of the genes that were selectively increased were associated with inflammatory gene ontology pathways (Fig. 2, C and D), suggesting that exercise promoted an elevation of inflammation in type 2 diabetes as compared with NGT. Gene ontology enrichment analysis performed on up- and down-regulated genes separately uncovered similar biological processes in NGT and type 2 diabetes post exercise, but different pathways were enriched after the recovery period (Fig. 2E). Four pathways related to inflammation (neutrophil activation, T cell activation, regulation of cell adhesion, and extracellular matrix organization), as well as pathways related to hypoxia (hemopoiesis) and oxidative stress, were selectively enriched in skeletal muscle of men with type 2 diabetes after recovery. Conversely, pathways enriched in genes associated with mitochondrial respiration were decreased in individuals with type 2 diabetes. Gene set enrichment analysis on transcripts ranked based on interaction effect (Exercise*T2D) demonstrated that the differential response to exercise in men with type 2 diabetes was mostly due to enriched expression of genes associated with extracellular matrix remodeling and inflammatory pathways, whereas genes associated with metabolism were diminished (Fig. 2, F and G).

The men with type 2 diabetes presented with lower maximal exercise workload and maximal oxygen uptake, indicating reduced overall physical fitness (table S1). To assess whether inflammatory responses were due to different training status between the diagnosis groups, cytokine induction was correlated to VO_2_ max and maximum workload. There were no significant correlations between cytokine induction and VO_2_ max (fig. S1A) or maximal workload (fig. S1B). In the MetaMEx database (*9*), the cytokine profile we observed in individuals with type 2 diabetes was not apparent in any other exercise study in healthy, nonathlete individuals. This cytokine profile was not detected even in studies of healthy individuals performing very intense exercise. Collectively, our results suggest that the inflammatory response observed in individuals with type 2 diabetes was not a consequence of lower fitness or exercise intensity compared with the individuals with NGT.

Exercise-induced changes in the inflammatory mRNA profile of skeletal muscle were not reflected systemically in plasma. Exercise increased alpha 2-macroglobin (A2M), C-reactive protein (CRP), haptoglobin, serum amyloid P component (SAP), and tissue plasminogen activator (tPA) in a similar manner in both diagnosis groups (fig. S2). Baseline haptoglobin and serum amyloid A (SAA) were increased, and fibrinogen levels were reduced in men with type 2 diabetes (fig. S2). Exercise did not alter levels of creatine kinase-muscle (CKM), chemokine (C-X-C motif) ligand 2 (CXCL2), or interleukin-6 (IL-6). Overall, there were no interaction effect of exercise and type 2 diabetes in plasma level of inflammatory markers, suggesting that the local inflammatory response was not due to either tissue damage or increased systemic inflammation.

### Local infiltration of skeletal muscle by immune cells

**Fig. 3.**
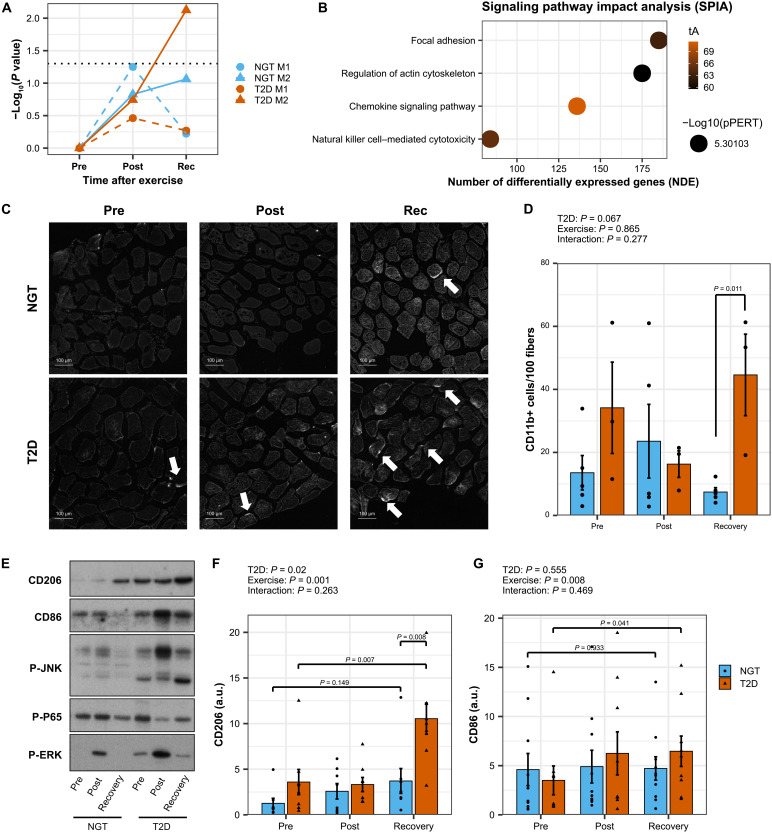
Elevated exercise-induced infiltration of skeletal muscle by immune cells in individuals with type 2 diabetes. (**A**) Overrepresentation analysis using Fisher’s exact test on pro- and anti-inflammatory genes signatures. (**B**) Signaling pathway impact analysis (SPIA). (**C**) Representative images of skeletal muscle labeled with CD11b. (**D**) Quantification of the number of immune cells per myofiber performed with ImageJ. Data are means ± SE and individual data points, *n* = 3 to 5. Two-way ANOVA (exercise and type 2 diabetes) and pairwise *t* tests. (**E**) Macrophage markers and inflammatory pathways analyzed by Western blotting. Representative blots. (**F** and **G**) Western blot quantifications. Data are means ± SE and individual data points, *n* = 8 to 10. Two-way ANOVA (exercise and type 2 diabetes) and pairwise *t* tests. a.u., arbitrary units.

Exercise induces an M2-like immune response associated with skeletal muscle hypertrophy and tissue remodeling (*10*, *11*). A curated database of M1 and M2 blood-derived macrophages was generated to define a transcriptomic signature of human M1 and M2 macrophage states (fig. S3A). Overrepresentation analysis using M1 and M2 signatures demonstrated an enhanced enrichment in M2-associated genes in men with type 2 diabetes after the recovery period (Fig. 3A). Signaling pathway impact analysis (SPIA) validated the activation of pathways associated with chemokine signaling and leukocyte adhesion in men with type 2 diabetes (Fig. 3B). This finding was confirmed by immunohistochemistry staining and Western blot analysis for immune cell markers in skeletal muscle. The number of cells expressing the general immune marker CD11b was increased in men with type 2 diabetes after the recovery period (Fig. 3, C and D, and fig. S3B). In agreement, the abundance of CD206, a marker for infiltrating macrophages, and CD86, a marker for all macrophages, was increased in skeletal muscle lysates from men with type 2 diabetes after the recovery period (Fig. 3, E to G). Stress kinases [extracellular signal–regulated kinase (ERK) and Janus kinase (JNK)] were phosphorylated immediately post exercise, but the response was not significantly different in individuals with type 2 diabetes (Fig. 3E). The canonical nuclear factor κB pathway was not activated, confirming that the attraction of immune cells was due to sterile inflammatory processes (Fig. 3E).

### Exercise-induced cytokine production in skeletal muscle from men with type 2 diabetes

**Fig. 4.**
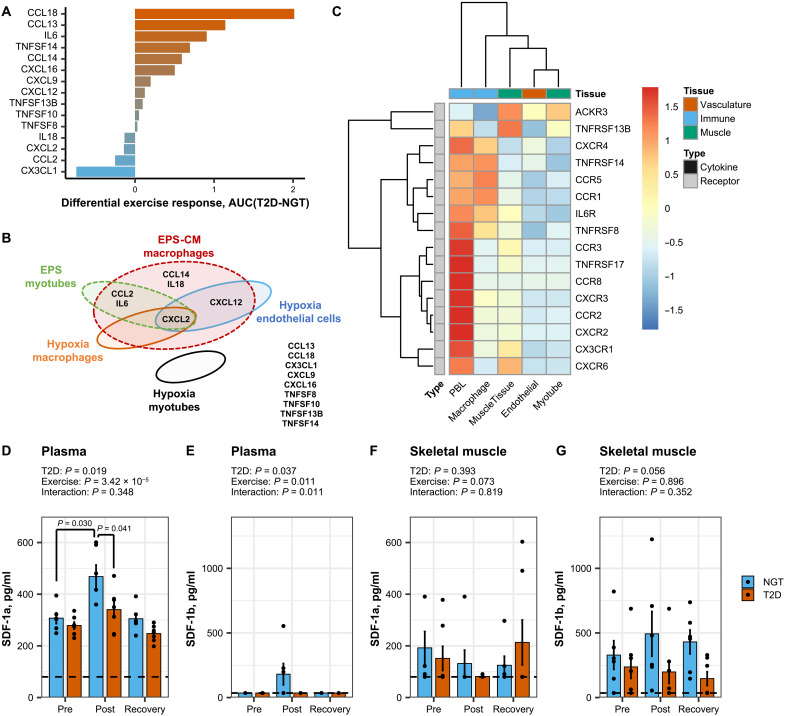
Cytokine cross-talk in skeletal muscle tissue. (**A**) Differential response in skeletal muscle from men with T2D versus NGT for exercise-responsive cytokines (FDR < 0.05). Area under the curve of mRNA to estimate the induction of genes compared with their relative baseline. Individual data for each cytokine in all conditions are available in fig. S2A. (**B**) Cytokines regulated by either EPS in primary human myotubes (green), hypoxia in macrophages (orange), or hypoxia in endothelial cells (blue). Cytokines induced in human THP1 macrophages in response to conditioned media from electrical pulse–stimulated myotubes (red). No cytokines were induced in human primary myotubes exposed to hypoxia (black). Several cytokines were not induced under any condition (side list). Individual data for each cytokine in all conditions are available in figs. S2 and S3. (**C**) Publicly available RNA sequencing data from bulk skeletal muscle tissue and peripheral blood leukocyte (PBL), as well as primary myotube and monocyte-derived macrophages differentiated in vitro. (**D** to **G**) CXCL12/SDF-1 measurement in plasma and skeletal muscle tissue lysate of men with T2D (*n* = 6) versus healthy individuals (*n* = 7). Alpha and beta isoforms of CXCL12 were quantified using enzyme-linked immunosorbent assay (ELISA). Dotted lines represent the detection threshold of the assays. Data are mean ± SE and individual datapoints, *n* = 6 to 7, two-way ANOVA (exercise and T2D) and pairwise *t* tests.

Cytokines are critical for immune responses, and 15 of these factors were regulated in skeletal muscle by exercise in men with either type 2 diabetes or NGT. The baseline (pre-exercise) expression of cytokines was not different between individuals with type 2 diabetes or NGT. The exercise-induced induction of most of these cytokines, excluding *CCL2*, *CXCL2*, *IL18*, and *CX3CL1*, was preferentially elevated in men with type 2 diabetes (Fig. 4A and fig. S4A). To decipher the specific contribution of myocytes to the production of exercise-responsive cytokines, primary skeletal muscle cells were derived from skeletal muscle biopsies obtained from individuals with type 2 diabetes or healthy controls. In primary myotubes submitted to electrical pulse stimulation (EPS), to mimic exercise in vitro, only *CCL2*, *CXCL2*, and *IL6* were affected (Fig. 4B and fig. S4B). In addition, stressed myotubes release noncytokine mediators, such as nucleotides or metabolites, which affect immune cell migration and the cellular phenotype (*12*). Thus, we tested conditioned medium from myotubes exposed to EPS for an ability to trigger the production of cytokines in human THP1 macrophages. *CCL2*, *CCL14*, *CXCL2*, *CXCL12*, *IL6*, and *IL18* levels were increased in macrophages in response to conditioned media from electrically stimulated myotubes (Fig. 4B and fig. S4C).

The induction of a local inflammatory response in men with type 2 diabetes after exercise may be due to physiological factors reflected in the gene ontology enrichment analysis (Fig. 2E), including response to heat, metabolic stress, oxidative stress, and hypoxia (“regulation of hemopoiesis”). These stress pathways were activated post exercise in both diagnosis groups, but “oxidative stress” and “hemopoiesis” remained elevated at the recovery time point only in men with type 2 diabetes. Cytokine production in skeletal muscle could therefore result directly from the contraction event, or from other factors including hypoxia, and subsequent activation of myocytes and the surrounding immune and vascular cells. However, mRNA levels of exercise-responsive cytokines were unaltered in myotubes exposed to hypoxia (1% oxygen) for up to 24 hours (Fig. 4B and fig. S5A). Overall, cytokine mRNA levels were not different in myotube cultures from individuals with type 2 diabetes as compared with NGT. Moreover, mRNA levels of many cytokines were undetectable, suggesting that inflammatory cytokine production in adult skeletal muscle in vivo is mainly due to nonmuscle cells (immune, endothelial, etc.). Publicly available data revealed that hypoxia selectively induced a subset of the exercise-responsive cytokines identified in humans. Hypoxia increased *CXCL2* mRNA expression in human blood–derived macrophages (fig. S5B) and *CXCL2* and *CXCL12* mRNA expression in human endothelial cells (fig. S5C). Collectively, experiments in myocytes, macrophage, and endothelial cells in vitro provide evidence to suggest that the inflammatory cytokine profile observed in skeletal muscle from individuals with type 2 diabetes after exercise arises from a complex interaction between several cell types, including endothelial, muscle, and immune cells in response to multiple stimuli including, but not limited to, contraction and hypoxia (Fig. 4B).

Further insight into the directionality of the communication between cell types was obtained by comparing the mRNA expression of cytokine receptors in adult skeletal muscle, primary myotubes, monocyte-derived macrophages, peripheral blood leukocytes, and endothelial cells (Fig. 4C). Most of the cytokine receptors were highly expressed on circulating immune cells and relatively lower in biopsies and myotubes, suggesting that many of the cytokines produced in skeletal muscle would target, attract, and activate immune cells. Only atypical chemokine receptor 3 also known as C-X-C chemokine receptor type 7 (CXCR-7) (*ACKR3/CXCR7*), a receptor for CXCL12, was highly expressed in myotubes as compared with immune cells, suggesting that CXCL12/SDF-1 (stromal cell-derived factor 1) may act on skeletal muscle cells.

The concentration of CXCL12/SDF-1α, but not CXCL12/SDF-1β, was elevated immediately post exercise in plasma from individuals with NGT (Fig. 4, D and E). In skeletal muscle tissue lysate, CXCL12/SDF-1α was undetectable in most samples (Fig. 4F), and the abundance CXCL12/SDF-1β did not change in response to exercise (Fig. 4G). CXCL2 and CX3CL1 were also increased by exercise, but CCL2, CXCL16, and CXCL9 were not affected (fig. S6). On the basis of mRNA and protein concentration in plasma and human skeletal muscle biopsies, cultures of primary human myotubes and macrophages, and the expression level of cytokines and receptors in skeletal muscle and immune and vascular cells, the chemokine CXCL12 was prioritized for further characterization as an exercise-responsive cytokine.

### Systemic hypoxia does not trigger cytokine production in skeletal muscle

**Fig. 5.**
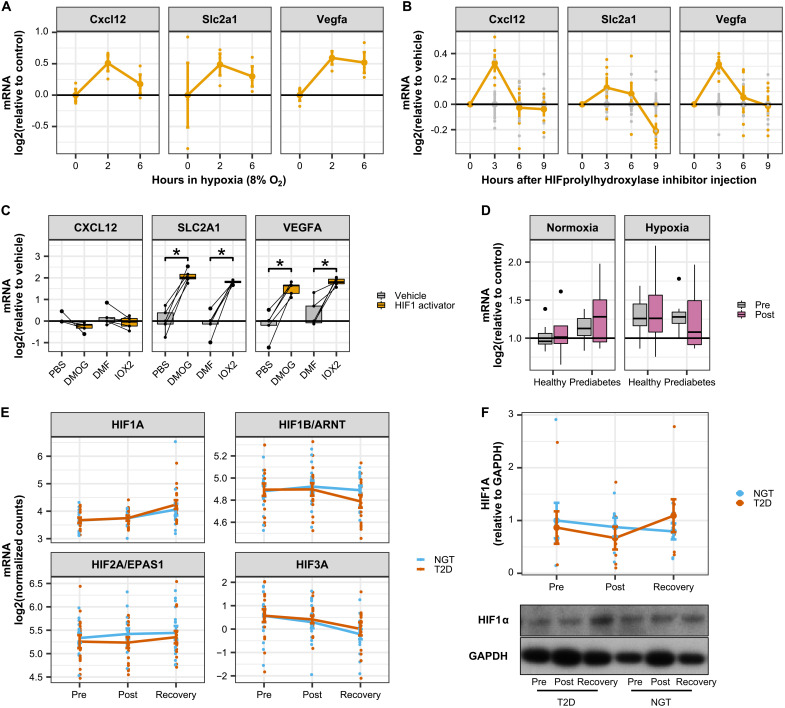
CXCL12 is induced by hypoxia in skeletal muscle tissue but not in primary myotube cultures. (**A**) Publicly available data (GSE81286) for cytokine mRNA expression in skeletal muscle from mice exposed to hypoxia (8% oxygen for up to 6 hours). Data are means ± SE. (**B**) Publicly available data (GSE95244) for cytokine mRNA expression in skeletal muscle from mice in which HIF1α was activated by an injection of an HIF prolyl hydroxylase inhibitor. Data are area under the curve of the time courses presented in fig. S3C. *P < 0.05 at least one time point after injection of the prolyl hydroxylase inhibitor. (**C**) Primary human skeletal muscle cells treated for 4 hours with the HIF1A stabilizers DMOG (100 μM) or IOX2 (200 μM). Box-and-whisper plots from five independent donors. *P < 0.05 paired *t* test versus respective vehicles. (**D**) CXCL12 mRNA expression in human skeletal muscle. Biopsies were collected before and after an acute bout of exercise in men with prediabetes versus metabolically healthy controls. Half of the participants performed exercise under hypoxia. Box-and-whisper plots from seven to eight independent donors. (**E**) HIF mRNA in skeletal muscle biopsies from men with type 2 diabetes (*n* = 20) versus healthy individuals (*n* = 18) from RNA sequencing data presented in Fig. 2. Data are means ± SE. (**F**) HIF1A protein abundance in skeletal muscle biopsies from men with type 2 diabetes (*n* = 7) versus healthy individuals (*n* = 7). Data are means ± SE.

To test whether hypoxia could be a causal mechanism in the production of cytokines, the mRNA expression of exercise-responsive cytokines, including *CXCL12*, was determined in publicly available datasets of murine models of hypoxia exposure (Fig. 5, A and B). In mouse skeletal muscle, mRNA expression of *Cxcl12* peaked 2 hours after hypoxia exposure (Fig. 5A) and 3 hours after injection of a hypoxia-inducible factor (HIF) prolyl hydroxylase inhibitor (Fig. 5B). While these latter effects were not significant after FDR correction, *Cxcl12* mRNA expression closely followed the profile of typical hypoxia-responsive genes, including Glucose transporter 1 (or GLUT1), also known as solute carrier family 2, facilitated glucose transporter member 1 (*SLC2A1*) and *VEGFA*. However, expression of *CXCL12* was unaffected in primary human myotubes exposed to HIF prolyl hydroxylase inhibitors [dimethyloxalylglycine (DMOG) and IOX2] despite GLUT1/*SLC2A1* and *VEFGA* mRNA being induced (Fig. 5C). Collectively, these results suggest that hypoxia triggers CXCL12 expression specifically in nonmuscle cells residing within skeletal muscle tissue.

Because systemic hypoxia may also affect the expression of cytokines in skeletal muscle, we measured *CXCL12* mRNA in skeletal muscle biopsies collected from individuals with either normal or impaired glucose tolerance after acute exercise under hypoxic conditions as described earlier (*13*). *CXCL12* mRNA was not altered by glucose tolerance, exercise, or hypoxia (Fig. 5D). Moreover, mRNA and protein levels of HIF1A were not differentially affected after exercise in skeletal muscle from individuals with type 2 diabetes as compared with NGT (Fig. 5, E and F). Collectively, these data show that systemic hypoxia does not induce CXCL12 mRNA in skeletal muscle. Therefore, local tissue hypoxia within skeletal muscle could contribute to an exercise-induced inflammatory response in individuals with type 2 diabetes, through a mechanism involving local and specific activation of nonmyocyte cells residing in skeletal muscle tissue.

### CXCL12 regulates skeletal muscle cell proliferation and differentiation

**Fig. 6.**
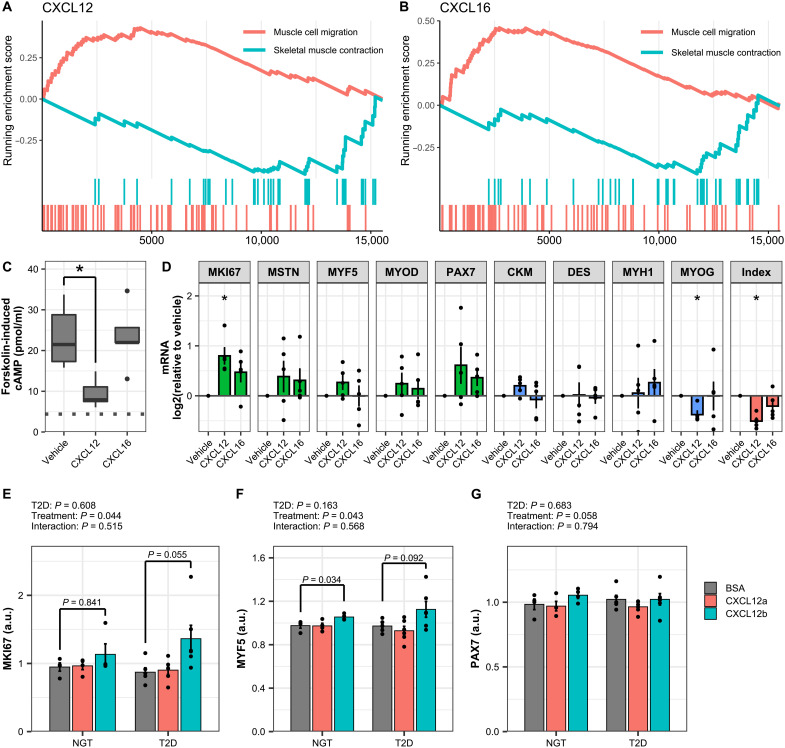
CXCL12 alters skeletal muscle cell differentiation. (**A** and **B**) Gene set enrichment analysis of genes correlated with CXCL12 and CXCL16 in the RNA sequencing of skeletal muscle biopsies. The pathway “muscle cell migration” was positively enriched, while “skeletal muscle contraction” was negatively enriched. (**C**) Inhibition of forskolin-induced cAMP production by CXCL12 or CXCL16 (100 ng/ml) measured in C2C12 myotubes as described in Materials and Methods. Box-and-whisper plots from five independent experiments. *P < 0.05. (**D**) C2C12 myoblasts were exposed to CXCL12 or CXCL16 (100 ng/ml) every other day for 5 days during the differentiation process as described in Materials and Methods. mRNA expression of genes markers of myoblasts or myotubes was measured by qPCR. Data are means ± SE, *n* = 5 independent experiments, repeated-measures ANOVA with Tukey’s posttest comparing cytokines to vehicle, *P < 0.05. (**E** to **G**) Primary human skeletal muscle cells from individuals with type 2 diabetes (T2D) or NGT were exposed to CXCL12a/SDF-1α or CXCL12b/SDF-1β (100 ng/ml) during the differentiation process as described in Materials and Methods. mRNA expression of gene markers of myoblasts or myotubes were measured by qPCR. Data are means ± SE, *n* = 4/6 cultures from independent donors (NGT/T2D).

CXCL12 was the only cytokine that showed a differential response to exercise (Fig. 4A), including indirect activation by EPS in macrophages, activation by hypoxia in endothelial cells, and expression of the receptor on primary human myotubes (Fig. 4C). Collectively, these observations suggest that CXCL12 may be produced by supporting cells during exercise and act on skeletal muscle to promote tissue remodeling. Gene set enrichment analysis of genes ranked by Spearman’s correlation demonstrated a positive association of CXCL12 and CXCL16 with “muscle cell migration” and a negative association with “skeletal muscle contraction” (Fig. 6, A and B). CXCL12 and CXCL16 were therefore prioritized for further validation for a functional role on proliferation and differentiation.

CXCL12 binds to the G protein–coupled receptors CXCR4 and ACKR3/CXCR7. Stimulation of C2C12 myotubes with CXCL12 inhibited forskolin-induced adenosine 3′,5′-monophosphate (cAMP) production, confirming that CXCL12 signals through a G protein–coupled receptor in skeletal muscle cells (Fig. 6E). Stimulation of C2C12 myoblasts during differentiation with CXCL12, but not CXCL16, increased mRNA expression of myoblast markers and decreased the levels of genes expressed in mature myotubes, such that the differentiation index was reduced (Fig. 6F). Similarly, exposure of primary human skeletal muscle during differentiation to the beta isoform of CXCL12 increased the mRNA expression of myoblast markers MKI67 (Marker Of Proliferation Ki-67), MYF5 (Myogenic Factor 5), PAX7 (Paired Box 7) (Fig. 6, G to I). Collectively, these results suggest that CXCL12 acts directly on myoblasts to increase proliferation and reduce differentiation into myotubes.

## DISCUSSION

Inflammation is associated with myopathies, metabolic disease, obesity, and cardiovascular diseases, but it is also a beneficial component required for myogenesis and remodeling of skeletal muscle after exercise (*14*). Here, we reveal that an acute bout of aerobic exercise leads to a selective and unconventional immune response in skeletal muscle from individuals with type 2 diabetes. Three hours after acute exercise, individuals with type 2 diabetes exhibited enhanced mRNA expression of immune markers, cytokines, and chemokines associated with an increased infiltration of immune cells in skeletal muscle. The chemokine CXCL12 was particularly dysregulated in skeletal muscle from individuals with type 2 diabetes, and exposure of cultured muscle cells to this exerkine directly triggered signaling events affecting proliferation and differentiation.

In response to exercise, a myriad of factors including local tissue hypoxia, increased blood flow, heat, elevated stress hormone levels, and release of circulating factors by immune cells and myocytes (myokines and exerkines) can collectively and independently activate the immune system. At the onset of an exercise bout, leukocytes rapidly increase in the circulation in an exercise intensity–dependent manner (*15*, *16*). Some of these immune cells infiltrate skeletal muscle, particularly after muscle-damaging exercise, which produces an acute and local inflammatory response essential for tissue repair, promoting proliferation and fusion of stem cells (*17*). However, the immune system can be activated in the absence of myofiber damage, for instance, by hypoxia, a potent activator of the immune system that promotes the expression of cytokines and chemokines, with the ultimate objective to promote tissue remodeling and enhance vascularization (*18*). Here, we provide evidence that the exercise-inflammation signature in individuals with type 2 diabetes is distinct from that of individuals with NGT. The transcriptomic response, and in particular the production of exerkines responsive to hypoxia, such as CXCL2 and CXCL12, suggests that the exercise-induced inflammation signature in individuals with type 2 diabetes may arise from insufficient local oxygenation of working skeletal muscle.

We identified CXCL12 as an exerkine released in the circulation after exercise and with enhanced mRNA induction in individuals with type 2 diabetes in skeletal muscle. CXCL12 was not induced by either EPS or hypoxia in primary human myotubes but was increased in endothelial cells exposed to hypoxia and in macrophages exposed to conditioned medium from contracted myotubes. A cross-talk between those different cell types is therefore the likely cause of greater CXCL12 expression in skeletal muscle tissue. Whole-body deletion of CXCL12 impairs angiogenesis during skeletal muscle regeneration (*19*), but CXCL12 muscle-specific knockout mice retain angiogenic properties (*20*), also suggesting that CXCL12 produced by nonmuscle cells is more relevant for skeletal muscle remodeling.

CXCL12 is a chemokine; thus, its local production in skeletal muscle from individuals with type 2 diabetes may be related to the attraction of neutrophils, monocytes, and lymphocytes to the tissue. However, in our study, exercise did not affect the expression of the CXCL12 receptors, CXCR4 and CXCR7, despite a clear infiltration of immune cells in skeletal muscle. The immune cells infiltrating skeletal muscle in individuals with type 2 diabetes are therefore not likely attracted by CXCL12. The low protein levels of cytokines detected by Luminex in skeletal muscle, and the absence of an increase in the number of cells expressing CXCR4/7, suggest that CXCL12 might be produced locally to stimulate cells resident to the tissue, such as muscle fibers, endothelial cells, and satellite cells, in a paracrine manner. CXCR4 is expressed in satellite cells and stem cells, which are chemoattracted and activated by CXCL12 (*21*, *22*).

CXCR4-deficient mice exhibit impaired myogenesis (*23*), and injection of CXCL12 in a mouse model of skeletal muscle damage increases fiber diameter and decreases fibrosis (*24*, *25*). In vitro studies do not decipher the physiological role of CXCL12, with results showing reduced myogenin expression (*26*), increased anabolism and myotube diameter (*27*), or increased migration and fusion to skeletal muscle fibers (*28*, *29*). We found that an exposure of myoblasts to CXCL12 during the differentiation process inhibited myoblast fusion and decreased the expression of differentiation markers. Thus, our data suggest that CXCL12 might favor proliferation over differentiation. Considering our data and evidence from mouse models (*23*–*25*), the induction of CXCL12 in skeletal muscle from individuals with type 2 diabetes is therefore likely a beneficial response, promoting local muscle stem cell recruitment and activation, leading to skeletal muscle remodeling.

Most individuals with type 2 diabetes are prescribed metformin as a first line of treatment, alone or in addition to lipid-lowering medication. Metformin was prescribed to the majority (80%) of the individuals with type 2 diabetes in the current study. Statin prescription was also more prevalent in individuals with type 2 diabetes. Participants abstained from medications 24 hours before the exercise bout, and we did not detect a systematic transcriptome difference introduced by statin or metformin use in principle component analyses. However, the current study was not designed to directly assess the effects of medication on the exercise responses and thus lacks statistical power to draw any firm conclusions related to the effects of metformin or statins on the transcriptomic or metabolic response, considering the relatively small number of subjects in each treatment group. We therefore cannot exclude the possibility that metformin or statin use influenced our results. Statins have been linked to muscle myalgia and myopathy (*30*), and metformin impairs skeletal muscle hypertrophy in response to resistance training (*31*), although it does not affect mitochondrial respiration, oxidative stress, or AMP-activated protein kinase (AMPK) activation (*32*). Further investigations are warranted to ascertain whether statins or metformin directly influences the immunometabolic response of skeletal muscle to acute exercise.

The balance between immune cell function and metabolism is paramount for proper tissue and whole-body health. Efforts are underway to understand the immunometabolic molecular events and signaling pathways that respond to diverse physiological stressors that alter nutrient and energy homeostasis. Our findings highlight the complexity of immunometabolic responses in skeletal muscle and the interactions between exercise-induced and metabolism-induced inflammation. We provide evidence that an acute bout of aerobic exercise triggers a distinct and unconventional inflammatory response in skeletal muscle from men with type 2 diabetes. Moreover, we identify CXCL12 as a plausible exerkine and mediator of skeletal muscle adaptation to exercise in individuals with type 2 diabetes. Given the health-promoting effects of regular physical exercise on metabolism and skeletal muscle function (*3*), and the evidence that nonsteroidal anti-inflammatory drugs blunt the beneficial effects of exercise on skeletal muscle remodeling (*33*, *34*), the exacerbated inflammatory response in individuals with type 2 diabetes is likely a beneficial response to acute exercise. Regular exercise training may help normalize this acute immunological response while concomitantly improving insulin sensitivity and reducing cardiometabolic risk (*35*). Our study highlights the role of inflammation and specifically the role of CXCL12 as an exerkine functioning as a positive effector in the adaptive response of skeletal muscle to acute exercise in metabolic diseases. This work underscores the potential for therapies targeting inflammation to improve the benefits of exercise in people with type 2 diabetes.

### Limitations of the study

Our study was limited to an acute study of middle-aged men with or without type 2 diabetes; thus, we did not specifically consider the impact of age, sex, or race in assessing the effects of acute exercise on the transcriptomic and immunometabolic responses. Inflammatory responses in individuals with type 2 diabetes were elevated 3 hours after exercise cessation, but our study did not investigate the consequences of inflammation days after the exercise bout. Last, our study was not designed to assess drug/exercise interactions (*36*), and we cannot exclude the possibility that prolonged use of prescription medicines may influence the baseline or exercise-induced transcriptomic or immunometabolic signatures.

## MATERIALS AND METHODS

### Human study design

The study was performed according to the Declaration of Helsinki, and all participants gave their informed consent. The ethical committees at the Karolinska Institutet and Umeå University approved the study protocols. The study was performed in Stockholm and Umeå (Sweden) on a total of 18 men with NGT and 20 men with type 2 diabetes. Clinical characteristics of the study participants are presented in table S1. A prescreening evaluation was performed, which included resting electrocardiography, a health interview, anthropometric measurements, and VO_2_ max testing, using a Rodby RE 820/830 bicycle ergometer. Metrics obtained from the VO_2_ max test were used to set the exercise intensity during the exercise visit. Exclusion criteria were blood pressure >160/95 mmHg, inability to perform cycling exercise, cardiovascular disease, smoking, and insulin treatment. Clinical parameters were compared using unpaired Students *t* test with a *P* value of <0.05 considered significant.

The participants returned 3 to 7 days after the screening visit for baseline (rest) measurements. All participants reported to the laboratory after an overnight fast and were instructed to refrain from taking medications 24 hours before the visit. Blood samples and vastus lateralis skeletal muscle biopsies (rest) were obtained as described (*37*). An oral glucose tolerance test was performed, and blood measurements were taken over 2 hours. Participants returned to the clinic 5 to 10 days after the baseline measurement and performed an acute bout of exercise on a cycle ergometer (Rodby). The workload was set to 85% of individually measured maximal heart rate, which was maintained for 30 min. Directly after the exercise bout, additional blood samples and skeletal muscle biopsies were taken. The participants then rested for 3 hours before the last blood samples and biopsy were taken from the contralateral leg.

### Primary cell culture

The ethical committee at Karolinska Institutet approved the protocols. Primary muscle cells were isolated from vastus lateralis biopsies derived from healthy men [age, 55 ± 7 years; body mass index (BMI), 24.4 ± 2.4 kg/m^2^]. In experiments comparing individuals with type 2 diabetes and NGT, primary myocytes were isolated from vastus lateralis biopsies from men (age, 50 ± 10 years; BMI, 22.9 ± 2.4 kg/m^2^) with or without a clinically diagnosis of type 2 diabetes.

### Cell lines

Mouse C2C12 muscle cells were obtained from the American Type Culture Collection (ATCC; CRL-1772). Cells were cultured and differentiated as described (*38*). THP1 human monocytes purchased from the ATCC were grown in RPMI 1640 containing 5% fetal bovine serum and supplemented with penicillin (100 U/ml), streptomycin (100 μg/ml), and amphotericin B (250 ng/ml). Differentiation was induced by diluting cells to 2.10^6^ cells/ml in medium containing phorbol 12-myristate 13-acetate (100 ng/ml). After 24 hours, cells were washed with phosphate-buffered saline and allowed to stabilize for 24 hours in regular growth medium before experiments. Differentiation was monitored under the microscope, and the absence of mycoplasma contamination was routinely confirmed by polymerase chain reaction (PCR).

### RNA isolation and RNA sequencing

RNA was extracted using the commercially available kit miRVana (Thermo Fisher Scientific) according to the manufacturer’s instructions. The quantity and quality of the RNA samples were assessed using BioAnalyzer, with a median RNA integrity value of 8.8. Samples were sent to the Swedish National Genomics Infrastructure facilities in Stockholm for library preparations and sequencing. Illumina RiboZero kit was used to create the libraries. Sequencing was performed using Illumina HiSeq 2500 high output V4, with a targeted depth of >20 million 125–base pair paired-ended reads per sample. RNA sequencing data have been deposited at Gene Expression Omnibus (GEO; GSE202295).

### RNA sequencing data analysis

Adapters were trimmed with Trim Galore! v0.6.4, and reads were mapped to Human GRCh38 reference genome assembly using STAR v2.6.1d. For a detailed description of the pipeline, see https://github.com/nf-core/rnaseq/blob/1.4.2/docs/output.md. Transcripts were filtered so that a minimum of *n* counts was detected in at least one of the six groups (NGT/type 2 diabetes, pre/post/rec), with *n* = (number of samples in the group)*10. Two samples were excluded because of poor correlation with other samples and clear separation in principal components analysis. An additional donor was then excluded because only the baseline sample could be analyzed. Differential expression analysis was performed with the edgeR package (*39*), with pairing per individual.

### Overrepresentation and gene set enrichment analyses

Overrepresentation analysis of gene ontology biological processes was performed using ClusterProfiler (*40*), using genes that passed the significance threshold FDR < 0.01. Gene set enrichment analysis was also performed using ClusterProfiler (*40*) on genes ranked based on log2(fold change). Gene ontology biological processes were considered significant at FDR < 0.05. SPIA was used to evaluate activation of Kyoto Encyclopedia of Genes and Genomes pathways (*41*).

### Blood measurements

Heparin plasma collected during the exercise day was used to investigate cytokine levels. Analysis was performed on a subset of the full cohort. IL-6, creatine, and CXCL2 levels were investigated using the following kits: human IL-6 ELISA MAX deluxe (BioLegend, #430505), human CKM ELISA kit (Abcam, ab185988), and human MIP2 ELISA kit (CXCL2) (Abcam, ab184862), according to the manufacturer’s instructions. A human acute phase 5-plex panel (Bio-Rad, #171a4008m, lot 5036639) was used to measure ferritin, fibrinogen, procalcitonin, serum amyloid A, and tissue plasminogen activator, with a sample dilution of 1:100. A human acute phase 4-plex panel (Bio-Rad, #171a4009m) was used to measure α-2-macroglobin, CRP, haptoglobin, and serum amyloid P, with a sample dilution of 1:10,000. CXCL12 was measured in plasma diluted 1:2 using SDF-1 alpha/CXCL12A human ELISA kit (Invitrogen, lot EHCXCL12A) and SDF-1 beta/CXCL12B human ELISA kit (Invitrogen, lot EHCXCL12B). All assays were performed according to the manufacturer’s instructions. Values below detection were imputed with the minimum concentration measurable by the kit according to the manufacturer. Blood measurements were tested for outliers using interquartile range and analyzed using two-way repeated analysis of variance (ANOVA) in R v4.0.4. *P* < 0.05 was considered significant.

### Immunofluorescence analysis

Analysis was performed on a subset of the full cohort. Participants were invited back to the laboratory on a separate occasion, and a skeletal muscle biopsy was taken for immunofluorescence staining. Biopsies were stored overnight in 4% formaldehyde in 0.1 M phosphate buffer solution. The following morning, they were placed in 10% sucrose in 0.1 M phosphate buffer solution for 2 to 10 days. After collecting biopsies from all time points, samples from an individual participant were placed in the same optimal cutting solution block and frozen on dry ice. Sections were cut with a thickness of 16 μm and incubated with primary antibodies for CD11b (#AMAb90911, Human Antibody Atlas), desmin (#PAB11453, Abnova), and von Willebrand factor (#AB7356, MerckMillipore) for 16 hours at 4°C. Sections were washed twice in tris-buffered saline with Tween-20 (TBST) for 15 min at room temperature before incubating with the secondary antibody mix for 90 min. Tissue autofluorescence was blocked using 1% Sudan Black (in 70% ethanol) and mounted with polyvinyl alcohol/glycerol containing 2.5% DABCO (Sigma-Aldrich). Images of the slides were acquired on an automated VSlide slide scanning system (MetaSystems, Altlussheim, Germany). Quantification of muscle fibers and immune cells was done using the “analyze particle” function in Fiji. Immunofluorescence data were tested for outliers using interquartile range and analyzed using two-way repeated ANOVA in R v4.0.4, with a significance threshold of *P* < 0.05.

### Cytokine measurement in skeletal muscle biopsies

Analysis was performed on a subset of the full cohort. Biopsy samples were homogenized using TissueLyzer (21 Hz, 1.5 min) in lysis buffer (Bio-Plex cell lysis kit, #171304011, according to the supplier’s instruction). Samples were frozen at −80°C for 2 hours and then thawed and centrifugated (4 min, 4500*g*) at 4°C. Supernatants were collected and protein concentration was measured using a bicinchoninic (BCA) assay. Samples were diluted to 3 μg/μl and stored at −80°C until further use. Cytokines were measured using a custom Bio-Plex Multiplex Immunoassay System (Bio-Rad) on samples diluted 1:4 in water according to the manufacturer’s instructions. CXCL12 was measured in skeletal muscle lysate diluted 1:5 using SDF-1 alpha/CXCL12A human ELISA kit (Invitrogen, EHCXCL12A) and SDF-1 beta/CXCL12B human ELISA kit (Invitrogen EHCXCL12B). Values below detection were imputed with the minimum concentration measurable by the kit according to the manufacturer.

### Electrical pulse stimulation

Fully differentiated myotubes were grown in six-well plates and exposed to EPS, using the C-Pace EP Culture Pacer (IonOptix, MA, USA), as described (*38*). Cells were pulsed at 40 V, 1 Hz, and 2-ms pulse duration for 3 hours, and RNA was extracted immediately after EPS.

### Hypoxia in vitro

Fully differentiated myotubes were grown in six-well plates and placed in a hypoxic chamber (1% O_2_ and 5% CO_2_) for 3, 6, or 24 hours. Cells were lysed in the hypoxic chamber, and RNA was extracted as described below.

### Effect of cytokines on myoblast differentiation

C2C12 myoblasts were cultured as described above but with differentiation medium supplemented with 100 μg/ml of recombinant cytokines prepared from fresh stocks before every media change (Peprotech #250-20B, #250-28, #300-28A, and #300-28B). Differentiation medium supplemented with recombinant cytokines was changed every other day, and cells were collected for RNA extraction after 5 to 6 days of differentiation for C2C12 and 8 days of differentiation for primary human cells.

### RNA extraction and qPCR

Cultured muscle cells were lysed, and RNA was extracted using the E.Z.N.A total RNA kit (Omega Bio-tek, Norcross, GA, USA), before concentration was determined through spectrophotometry. All equipment, software, and reagents for performing the reverse transcription and quantitative PCR (qPCR) were from Thermo Fisher Scientific. Complementary DNA (cDNA) synthesis was performed from ~0.1 to 1 μg of RNA using random hexamers and the high-capacity cDNA reverse transcription kit, according to the manufacturer’s instructions. qPCR was performed on a StepOne Plus or Viia7 system. Relative gene expression was calculated by the comparative ΔΔ*C*_t_ method. For THP1 macrophages, the geometric mean of 18*S*, HPRT1 (hypoxanthine phosphoribosyltransferase 1), and *CYCS* (*Cytochrome C*, *Somatic*) was used as housekeeping reference. For human myotubes, the geometric mean of 18*S*, RPLPO (Large Ribosomal Protein), HPRT1, and TATA box–binding protein was used as housekeeping reference. For C2C12 cells, the geometric mean of 18*S*, HPRT, and PGK1 (phosphoglycerate kinase 1) was used as housekeeping reference.

### Protein extraction and immunoblot analysis

Cells were lysed in homogenization buffer (137 mM NaCl, 2.7 mM KCl, 1 mM MgCl_2_, 0.5 mM Na_3_VO_4_, 1% Triton X-100, 10% glycerol, 20 mM tris at pH 7.8, 10 mM NaF, 1 mM EDTA, 0.2 mM phenylmethylsulfonyl fluoride, and 1% protease inhibitor cocktail; Merck, Darmstadt, Germany). Homogenates were rotated for 40 min at 4°C and subjected to centrifugation (10 000*g* for 10 min at 4°C). Protein content of the supernatants was measured by BCA protein assay kit (Pierce Biotechnology, Rockford, IL, USA). Samples were prepared for SDS–polyacrylamide gel electrophoresis with Laemmli buffer [60 mM tris at pH 6.8, 2% (w/v) SDS, 10% (v/v) glycerol, 0.01% (w/v) bromophenol blue, and 1.25% (v/v) β-mercaptoethanol]. Equal amounts of protein were loaded and separated on Criterion XT Bis-Tris Gels (Bio-Rad, Hercules, CA, USA) and transferred to polyvinylidene fluoride membranes (Merck). Membranes were then stained with Ponceau S to confirm the quality of the transfer and equal loading of samples. Membranes were blocked with 5% nonfat milk in TBST (20 mM tris·HCl at pH 7.6, 137 mM NaCl, and 0.02% Tween-20) for 1 hour at room temperature and subsequently incubated overnight at 4°C with primary antibodies diluted in TBS with 0.1% (w/v) bovine serum albumin and 0.1% (w/v) NaN_3_. Membranes were washed with TBST and incubated with species-appropriate horseradish peroxidase–conjugated secondary antibody (1:25,000 in TBST with 5% nonfat milk). Proteins were then visualized by enhanced chemiluminescence (Amersham ECL Western Blotting Detection Reagent, Little Chalfont, UK). Protein content was quantified by densitometry (ImageLab, Bio-Rad). Antibodies against HIF1A (Santa Cruz Biotechnology, sc-13515), glyceraldehyde-3-phosphate dehydrogenase (Santa Cruz Biotechnology, sc-47724), phospho-p44/42 MAPK (Erk1/2) (Thr^202^/Tyr^204^) (Cell Signaling, #9101), phospho-SAPK/JNK (Thr^183^/Tyr^185^) (Cell Signaling, #4668), phospho-NK-kB p65 (Cell Signaling, #3033), CD206 (Cell Signaling, #24595), and CD86 (Cell Signaling, #91882) were used for the Western blot analysis.

### Exercise timeline from MetaMEx

Using the MetaMEx database (*9*), studies of acute exercise were selected, excluding athletes and individuals with chronic diseases or obesity. Studies were annotated with the time of biopsy collection after exercise cessation in the following categories: 0 to 1, 2 to 3, 4 to 6, 24, and 48 hours. Genes with more than 20% missing values across all studies were excluded. The average pre-/post-biopsy data for each study were extracted, and a linear model was calculated using empirical Bayes statistics for differential expression with the limma package (*42*). The analysis was performed to compare the effect of each time point to the pre-exercise samples in a paired setup (pre/post for each study), blocking for age, sex, sedentary status, and weight. After correction for multiple testing using Benjamini and Hochberg FDR, genes were considered significant at FDR < 0.05. Gene set enrichment analysis was performed on the basis of genes ranked on log2(fold change) using clusterProfiler (*40*). Gene ontology biological processes were considered significant at FDR < 0.05.

### M1/M2 macrophage signatures

The GEO repository was used to identify transcriptomic data of human primary blood–derived macrophages differentiated in vitro and polarized into M1 (with lipopolysaccharide, interferon-γ, or both) or M2 (with IL-4, IL-13, or both). Eight RNA sequencing datasets and 13 microarray datasets were found for a total *n* = 57 M0, *n* = 92 M1, and *n* = 87 M2 macrophages. All data were downloaded and processed in unison using empirical Bayes statistics for differential expression with the limma package (*42*), with blocking for differences between studies (fig. S1A). Genes were considered specific to the M1 or M2 polarization states if FDR < 10^−3^ and log2(fold change) > 0 compared with untreated (M0) and FDR < 10^−3^ and log2(fold change) > 2 comparing M1 and M2. The gene signature lists were used to test for enrichment against the human RNA sequencing data using Fisher’s exact test.

### Cytokine receptor expression in cells and tissues

Raw counts of RNA sequencing data were mined using ARCHS4 (*43*) for peripheral blood leukocytes, endothelial cells, skeletal muscle tissue, and primary myotube and monocyte-derived macrophages differentiated in vitro. Samples with less than 15,000 genes detected were excluded, and the data were filtered to have at least *n* counts in at least one of the tissue/cell groups with *n* = (number of samples)*5. Raw counts were normalized using variance stabilizing transformation with the DESeq2 package (*44*).

### Acute hypoxia and activation of HIF1a in mouse skeletal muscle

The GSE81286 dataset compared mouse skeletal muscle mRNA profile at baseline and after 2- or 6-hour exposure to 8% oxygen. The GSE95244 dataset contains data from skeletal muscle of mice injected with a prolyl hydroxylase inhibitor to activate HIF1A. Data were downloaded with GEOquery, normalized, and analyzed using empirical Bayes statistics for differential expression with the limma package (*42*).

### Acute hypoxia in primary endothelial cells and blood-derived macrophages

The GEO repository was used to identify transcriptomic data of human primary blood–derived macrophages (5 datasets) and primary endothelial cells (12 datasets) exposed to hypoxia. All data were downloaded and processed in unison using empirical Bayes statistics for differential expression with the limma package (*42*), with blocking for differences between studies (fig. S3, A and B).

### Primary human skeletal muscle cell differentiation

The GSE55650 and GSE166502 were downloaded and processed using standard pipelines for RNA sequencing (edgeR) or microarrays (RMA). Datasets were pooled and normalized using quantile normalization. Differences due to the different platforms used were accounted for by blocking in the linear model. Statistics were calculated with empirical Bayes statistics for differential expression with the limma package (*42*) and corrected for multiple comparisons with FDR (Benjamini and Hochberg).

### Statistical analysis

Analyses were performed using either R 4.0.2 (www.r-project.org) or GraphPad Prism 9.0 software (GraphPad Software Inc.). Comparisons were considered statistically significant at *P* < 0.05. Data are presented as box-and-whisker plots unless otherwise specified in figure legends. Normality was tested using the Shapiro-Wilk test before applying appropriate parametric or nonparametric tests. Statistical tests used are described in the figure legends. Bioinformatic analyses are described in their respective Materials and Methods sections. Data were corrected for multiple testing with FDR (Benjamini and Hochberg).

## Notes

View/request a protocol for this paper from *Bio-protocol*.

## References

[1] G. D. Cartee, R. T. Hepple, M. M. Bamman, J. R. Zierath, Exercise promotes healthy aging of skeletal muscle. Cell Metab. 23, 1034–1047 (2016). 27304505 10.1016/j.cmet.2016.05.007 PMC5045036

[2] L. Nesti, N. R. Pugliese, P. Sciuto, A. Natali, Type 2 diabetes and reduced exercise tolerance: A review of the literature through an integrated physiology approach. Cardiovasc. Diabetol. 19, 134 (2020). 32891175 10.1186/s12933-020-01109-1 PMC7487838

[3] M. Savikj, J. R. Zierath, Train like an athlete: Applying exercise interventions to manage type 2 diabetes. Diabetologia 63, 1491–1499 (2020). 32529411 10.1007/s00125-020-05166-9 PMC7351814

[4] S. R. Colberg, R. J. Sigal, J. E. Yardley, M. C. Riddell, D. W. Dunstan, P. C. Dempsey, E. S. Horton, K. Castorino, D. F. Tate, Physical activity/exercise and diabetes: A position statement of the American Diabetes Association. Diabetes Care 39, 2065–2079 (2016). 27926890 10.2337/dc16-1728 PMC6908414

[5] R. Mancilla, B. Brouwers, V. B. Schrauwen-Hinderling, M. K. C. Hesselink, J. Hoeks, P. Schrauwen, Exercise training elicits superior metabolic effects when performed in the afternoon compared to morning in metabolically compromised humans. Physiol. Rep. 8, e14669 (2021). 33356015 10.14814/phy2.14669 PMC7757369

[6] M. Savikj, B. M. Gabriel, P. S. Alm, J. Smith, K. Caidahl, M. Björnholm, T. Fritz, A. Krook, J. R. Zierath, H. Wallberg-Henriksson, Afternoon exercise is more efficacious than morning exercise at improving blood glucose levels in individuals with type 2 diabetes: A randomised crossover trial. Diabetologia 62, 233–237 (2019). 30426166 10.1007/s00125-018-4767-z PMC6323076

[7] B. Egan, J. R. Zierath, Exercise metabolism and the molecular regulation of skeletal muscle adaptation. Cell Metab. 17, 162–184 (2013). 23395166 10.1016/j.cmet.2012.12.012

[8] B. H. Goodpaster, L. M. Sparks, Metabolic flexibility in health and disease. Cell Metab. 25, 1027–1036 (2017). 28467922 10.1016/j.cmet.2017.04.015 PMC5513193

[9] N. J. Pillon, B. M. Gabriel, L. Dollet, J. A. B. Smith, L. Sardon Puig, J. Botella, D. J. Bishop, A. Krook, J. R. Zierath, Transcriptomic profiling of skeletal muscle adaptations to exercise and inactivity. Nat. Commun. 11, 470 (2020). 31980607 10.1038/s41467-019-13869-w PMC6981202

[10] S. M. Jensen, C. J. L. Bechshøft, M. F. Heisterberg, P. Schjerling, J. L. Andersen, M. Kjaer, A. L. Mackey, Macrophage subpopulations and the acute inflammatory response of elderly human skeletal muscle to physiological resistance exercise. Front. Physiol. 11, 811 (2020). 32792975 10.3389/fphys.2020.00811 PMC7393256

[11] R. G. Walton, K. Kosmac, J. Mula, C. S. Fry, B. D. Peck, J. S. Groshong, B. S. Finlin, B. Zhu, P. A. Kern, C. A. Peterson, Human skeletal muscle macrophages increase following cycle training and are associated with adaptations that may facilitate growth. Sci. Rep. 9, 969 (2019). 30700754 10.1038/s41598-018-37187-1 PMC6353900

[12] N. J. Pillon, Y. E. Li, L. N. Fink, J. T. Brozinick, A. Nikolayev, M. S. Kuo, P. J. Bilan, A. Klip, Nucleotides released from palmitate-challenged muscle cells through pannexin-3 attract monocytes. Diabetes 63, 3815–3826 (2014). 24917574 10.2337/db14-0150

[13] E. De Groote, F. A. Britto, E. Balan, G. Warnier, J. P. Thissen, H. Nielens, L. Sylow, L. Deldicque, Effect of hypoxic exercise on glucose tolerance in healthy and prediabetic adults. Am. J. Physiol. Endocrinol. Metab. 320, E43–E54 (2021). 33103453 10.1152/ajpendo.00263.2020

[14] O. Neubauer, S. Sabapathy, K. J. Ashton, B. Desbrow, J. M. Peake, R. Lazarus, B. Wessner, D. Cameron-Smith, K. H. Wagner, L. J. Haseler, A. C. Bulmer, Time course-dependent changes in the transcriptome of human skeletal muscle during recovery from endurance exercise: From inflammation to adaptive remodeling. J. Appl. Physiol. (1985) 116, 274–287 (2014). 24311745 10.1152/japplphysiol.00909.2013

[15] C. A. M. Gonçalves, P. M. S. Dantas, I. K. Dos Santos, M. Dantas, D. C. P. da Silva, B. Cabral, R. O. Guerra, G. B. C. Júnior, Effect of acute and chronic aerobic exercise on immunological markers: A systematic review. Front. Physiol. 10, 1602 (2019). 32038286 10.3389/fphys.2019.01602 PMC6993577

[16] P. Neves, T. Tenório, T. A. Lins, M. T. C. Muniz, T. C. Pithon-Curi, J. P. Botero, W. L. Do Prado, Acute effects of high- and low-intensity exercise bouts on leukocyte counts. J. Exerc. Sci. Fit. 13, 24–28 (2015). 29541095 10.1016/j.jesf.2014.11.003 PMC5812872

[17] J. M. Peake, O. Neubauer, P. A. Della Gatta, K. Nosaka, Muscle damage and inflammation during recovery from exercise. J. Appl. Physiol. (1985) 122, 559–570 (2017). 28035017 10.1152/japplphysiol.00971.2016

[18] H. K. Eltzschig, P. Carmeliet, Hypoxia and inflammation. N. Engl. J. Med. 364, 656–665 (2011). 21323543 10.1056/NEJMra0910283 PMC3930928

[19] D. Hardy, M. Fefeu, A. Besnard, D. Briand, P. Gasse, F. Arenzana-Seisdedos, P. Rocheteau, F. Chrétien, Defective angiogenesis in CXCL12 mutant mice impairs skeletal muscle regeneration. Skelet. Muscle 9, 25 (2019). 31533830 10.1186/s13395-019-0210-5 PMC6751827

[20] M. Yamada, C. Hokazono, K. Tokizawa, S. Marui, M. Iwata, V. A. Lira, K. Suzuki, S. Miura, K. Nagashima, M. Okutsu, Muscle-derived SDF-1α/CXCL12 modulates endothelial cell proliferation but not exercise training-induced angiogenesis. Am. J. Physiol. Regul. Integr. Comp. Physiol. 317, R770–R779 (2019). 31577158 10.1152/ajpregu.00155.2019

[21] C. C. Maesner, A. E. Almada, A. J. Wagers, Established cell surface markers efficiently isolate highly overlapping populations of skeletal muscle satellite cells by fluorescence-activated cell sorting. Skelet. Muscle 6, 35 (2016). 27826411 10.1186/s13395-016-0106-6 PMC5100091

[22] M. Z. Ratajczak, M. Majka, M. Kucia, J. Drukala, Z. Pietrzkowski, S. Peiper, A. Janowska-Wieczorek, Expression of functional CXCR4 by muscle satellite cells and secretion of SDF-1 by muscle-derived fibroblasts is associated with the presence of both muscle progenitors in bone marrow and hematopoietic stem/progenitor cells in muscles. Stem Cells 21, 363–371 (2003). 12743331 10.1634/stemcells.21-3-363

[23] V. Odemis, E. Lamp, G. Pezeshki, B. Moepps, K. Schilling, P. Gierschik, D. R. Littman, J. Engele, Mice deficient in the chemokine receptor CXCR4 exhibit impaired limb innervation and myogenesis. Mol. Cell. Neurosci. 30, 494–505 (2005). 16198599 10.1016/j.mcn.2005.07.019

[24] E. Brzoska, M. Kowalewska, A. Markowska-Zagrajek, K. Kowalski, K. Archacka, M. Zimowska, I. Grabowska, A. M. Czerwińska, M. Czarnecka-Góra, W. Stremińska, K. Jańczyk-Ilach, M. A. Ciemerych, Sdf-1 (CXCL12) improves skeletal muscle regeneration via the mobilisation of Cxcr4 and CD34 expressing cells. Biol. Cell 104, 722–737 (2012). 22978573 10.1111/boc.201200022

[25] Y. Maeda, Y. Yonemochi, Y. Nakajyo, H. Hidaka, T. Ikeda, Y. Ando, CXCL12 and osteopontin from bone marrow-derived mesenchymal stromal cells improve muscle regeneration. Sci. Rep. 7, 3305 (2017). 28607396 10.1038/s41598-017-02928-1 PMC5468354

[26] V. Odemis, K. Boosmann, M. T. Dieterlen, J. Engele, The chemokine SDF1 controls multiple steps of myogenesis through atypical PKCzeta. J. Cell Sci. 120, 4050–4059 (2007). 17971416 10.1242/jcs.010009

[27] M. Puchert, V. Adams, A. Linke, J. Engele, Evidence for the involvement of the CXCL12 system in the adaptation of skeletal muscles to physical exercise. Cell. Signal. 28, 1205–1215 (2016). 27237374 10.1016/j.cellsig.2016.05.019

[28] G. U. Bae, U. Gaio, Y. J. Yang, H. J. Lee, J. S. Kang, R. S. Krauss, Regulation of myoblast motility and fusion by the CXCR4-associated sialomucin, CD164. J. Biol. Chem. 283, 8301–8309 (2008). 18227060 10.1074/jbc.M706730200 PMC2276390

[29] K. Kowalski, A. Kołodziejczyk, M. Sikorska, J. Płaczkiewicz, P. Cichosz, M. Kowalewska, W. Stremińska, K. Jańczyk-Ilach, M. Koblowska, A. Fogtman, R. Iwanicka-Nowicka, M. A. Ciemerych, E. Brzoska, Stem cells migration during skeletal muscle regeneration–The role of Sdf-1/Cxcr4 and Sdf-1/Cxcr7 axis. Cell Adh. Migr. 11, 384–398 (2017). 27736296 10.1080/19336918.2016.1227911 PMC5569967

[30] J. R. Guyton, H. E. Bays, S. M. Grundy, T. A. Jacobson; The National Lipid Association Statin Intolerance Panel, An assessment by the Statin Intolerance Panel: 2014 update. J. Clin. Lipidol. 8, S72–S81 (2014). 24793444 10.1016/j.jacl.2014.03.002

[31] R. G. Walton, C. M. Dungan, D. E. Long, S. C. Tuggle, K. Kosmac, B. D. Peck, H. M. Bush, A. G. Villasante Tezanos, G. McGwin, S. T. Windham, F. Ovalle, M. M. Bamman, P. A. Kern, C. A. Peterson, Metformin blunts muscle hypertrophy in response to progressive resistance exercise training in older adults: A randomized, double-blind, placebo-controlled, multicenter trial: The MASTERS trial. Aging Cell 18, e13039 (2019). 31557380 10.1111/acel.13039 PMC6826125

[32] N. S. Pilmark, L. Oberholzer, J. F. Halling, J. M. Kristensen, C. P. Bønding, I. Elkjær, M. Lyngbæk, G. Elster, C. Siebenmann, N. F. Holm, J. B. B. Birk, E. L. Larsen, A. K. Meinild-Lundby, J. F. Wojtaszewski, H. Pilegaard, H. Poulsen, B. K. Pedersen, K. B. Hansen, K. Karstoft, Skeletal muscle adaptations to exercise are not influenced by metformin treatment in humans: Secondary analyses of two randomised, clinical trials. Appl. Physiol. Nutr. Metab. 47, 309–320 (2021). 34784247 10.1139/apnm-2021-0194

[33] M. Lilja, M. Mandic, W. Apro, M. Melin, K. Olsson, S. Rosenborg, T. Gustafsson, T. R. Lundberg, High doses of anti-inflammatory drugs compromise muscle strength and hypertrophic adaptations to resistance training in young adults. Acta Physiol (Oxf.) 222, 1–16 (2018). 10.1111/apha.12948 28834248

[34] T. A. Trappe, F. White, C. P. Lambert, D. Cesar, M. Hellerstein, W. J. Evans, Effect of ibuprofen and acetaminophen on postexercise muscle protein synthesis. Am. J. Physiol. Endocrinol. Metab. 282, E551–E556 (2002). 11832356 10.1152/ajpendo.00352.2001

[35] R. J. Verheggen, F. Poelkens, S. H. Roerink, R. E. Ramakers, M. Catoire, A. R. Hermus, D. H. Thijssen, M. T. Hopman, Exercise improves insulin sensitivity in the absence of changes in cytokines. Med. Sci. Sports Exerc. 48, 2378–2386 (2016). 27414688 10.1249/MSS.0000000000001035

[36] B. F. Miller, J. P. Thyfault, Exercise-pharmacology interactions: Metformin, statins, and healthspan. Physiology (Bethesda) 35, 338–347 (2020). 32783612 10.1152/physiol.00013.2020 PMC7642846

[37] M. E. Osler, T. Fritz, K. Caidahl, A. Krook, J. R. Zierath, H. Wallberg-Henriksson, Changes in gene expression in responders and nonresponders to a low-intensity walking intervention. Diabetes Care 38, 1154–1160 (2015). 25795414 10.2337/dc14-2606

[38] A. M. Abdelmoez, L. Sardón Puig, J. A. B. Smith, B. M. Gabriel, M. Savikj, L. Dollet, A. V. Chibalin, A. Krook, J. R. Zierath, N. J. Pillon, Comparative profiling of skeletal muscle models reveals heterogeneity of transcriptome and metabolism. Am. J. Physiol. Cell Physiol. 318, C615–C626 (2020). 31825657 10.1152/ajpcell.00540.2019 PMC7099524

[39] M. D. Robinson, D. J. McCarthy, G. K. Smyth, edgeR: A bioconductor package for differential expression analysis of digital gene expression data. Bioinformatics 26, 139–140 (2010). 19910308 10.1093/bioinformatics/btp616 PMC2796818

[40] G. Yu, L. G. Wang, Y. Han, Q. Y. He, clusterProfiler: An R package for comparing biological themes among gene clusters. Omics 16, 284–287 (2012). 22455463 10.1089/omi.2011.0118 PMC3339379

[41] A. L. Tarca, S. Draghici, P. Khatri, S. S. Hassan, P. Mittal, J. S. Kim, C. J. Kim, J. P. Kusanovic, R. Romero, A novel signaling pathway impact analysis. Bioinformatics 25, 75–82 (2009). 18990722 10.1093/bioinformatics/btn577 PMC2732297

[42] M. E. Ritchie, B. Phipson, D. Wu, Y. Hu, C. W. Law, W. Shi, G. K. Smyth, *limma* powers differential expression analyses for RNA-sequencing and microarray studies. Nucleic Acids Res. 43, e47 (2015). 25605792 10.1093/nar/gkv007 PMC4402510

[43] A. Lachmann, D. Torre, A. B. Keenan, K. M. Jagodnik, H. J. Lee, L. Wang, M. C. Silverstein, A. Ma’ayan, Massive mining of publicly available RNA-seq data from human and mouse. Nat. Commun. 9, 1366 (2018). 29636450 10.1038/s41467-018-03751-6 PMC5893633

[44] M. I. Love, W. Huber, S. Anders, Moderated estimation of fold change and dispersion for RNA-seq data with DESeq2. Genome Biol. 15, 550 (2014). 25516281 10.1186/s13059-014-0550-8 PMC4302049

