# Supplementary material for: Distinctive exercise-induced inflammatory response and exerkine induction in skeletal muscle of people with type 2 diabetes
Source: Sci Adv. 2022 Sep 7;8(36):eabo3192. doi: 10.1126/sciadv.abo3192 (PMC9451165; doi:10.1126/sciadv.abo3192)
Supplement: Supplementary file 1 — Table S1 Figs. S1 to S6 [file sciadv.abo3192_sm.pdf]

Science Advances
AAAS

Supplementary Materials for

Distinctive exercise-induced inflammatory response and exerkine induction in skeletal muscle of people with type 2 diabetes

Nicolas J. Pillon *et al.*

Corresponding author: Juleen R. Zierath, juleen.zierath@ki.se

Sci. Adv. **8**, eabo3192 (2022)
DOI: 10.1126/sciadv.abo3192

This PDF file includes:

Table S1
Figs. S1 to S6

	NGT	T2D	P VALUE
Age	59.9 ± 6.0	60.0 ± 4.8	0.595
BMI [kg/m2]	27.5 ± 2.8	28.3 ± 2.7	0.518
HbA1c [mmol/mol]	35.4 ± 3.4	47.1 ± 8.4	<0.001
Weight [kg]	87.9 ± 9.8	88.8 ± 13.6	0.988
Length [m]	1.79 ± 0.07	1.77 ± 0.08	0.471
Waist [cm]	98.2 ± 8.5	103.3 ± 8.2	0.106
Hip [cm]	101.9 ± 6.5	102.1 ± 9.1	0.489
W/H ratio	0.97 ± 0.05	1.01 ± 0.06	0.017
Body fat [%]	28.6 ± 5.8	30.4 ± 4.7	0.347
Fat mass [kg]	25.2 ± 6.6	26.9 ± 6.8	0.597
Lean mass [kg]	59.9 ± 5.7	59.3 ± 8.9	0.814
BP Systolic [mm Hg]	130.4 ± 13.2	133.8 ± 14.1	0.49
BP Diastolic [mmHg]	79.2 ± 7.1	78.7 ± 8.3	0.628
Pulse [BPM]	59.1 ± 8.1	68.6 ± 8.9	0.003
P-glucose 0 OGTT [mmol/L]	5.2 ± 0.5	7.2 ± 1.6	<0.001
P-glucose 30 OGTT [mmol/L]	8.0 ± 1.6	17.9 ± 23.9	<0.001
P-glucose 60 OGTT [mmol/L]	7.8 ± 2.2	15.7 ± 3.5	<0.001
P-glucose 120 OGTT [mmol/L]	5.4 ± 1.4	13.9 ± 3.1	<0.001
S-insulin 0 OGTT [mIE/L]	6.4 ± 3.5	11.5 ± 5.1	0.002
S-insulin 30 OGTT [mIE/L]	65.6 ± 48.9	42.5 ± 15.5	0.247
S-insulin 60 OGTT [mIE/L]	75.6 ± 57.0	58.5 ± 38.2	0.489
S-insulin 120 OGTT [mIE/L]	42.2 ± 47.1	66.1 ± 52.6	0.033
P-glucose 0 Exercise [mmol/L]	5.0 ± 0.8	8.0 ± 1.8	<0.001
P-glucose 35 Exercise [mmol/L]	5.5 ± 1.0	7.6 ± 1.5	<0.001
P-glucose 3h35 Exercise [mmol/L]	5.0 ± 0.6	6.8 ± 1.3	<0.001
S-insulin 0 Exercise [mIE/L]	10.9 ± 7.3	20.8 ± 9.9	0.001
S-insulin 35 Exercise [mIE/L]	9.2 ± 4.8	15.1 ± 7.7	0.014
S-insulin 3h35 Exercise [mIE/L]	5.6 ± 3.8	10.3 ± 5.3	0.004
P-Creatinine [µmol/L]	81.9 ± 12.9	78.4 ± 12.0	0.499
P-ASAT [µkat/L]	0.43 ± 0.12	0.50 ± 0.18	0.402
P-ALAT [µkat/L]	0.38 ± 0.10	0.58 ± 0.38	0.006
P-TG [mmol/L]	1.07 ± 0.44	1.36 ± 0.73	0.163
P-Chol [mmol/L]	5.4 ± 0.8	3.97 ± 0.83	<0.001
P-HDL [mmol/L]	1.44 ± 0.40	1.14 ± 0.26	0.015
P-LDL [mmol/L]	3.44 ± 0.78	2.20 ± 0.70	<0.001
S-C-peptide [nmol/L]	0.63 ± 0.26	0.99 ± 0.29	<0.001
VO2max [ml/kg/min]	36.9 ± 8.5	31.6 ± 8.3	0.078
Max workload [Watt]	247.4 ± 47.7	200.0 ± 49.8	0.013
Max heartrate [beats/min]	165.2 ± 9.2	166.0 ± 15.7	0.669
HOMA1-IR	1.50 ± 0.89	3.66 ± 2.02	<0.001
Metformin (Yes/No)	0/17	17/04	
Lipid lowering medication (Yes/No)	15/02	14/07	

Supplemental Table S1. Clinical parameters of participants included in the study. Data are mean ± SD

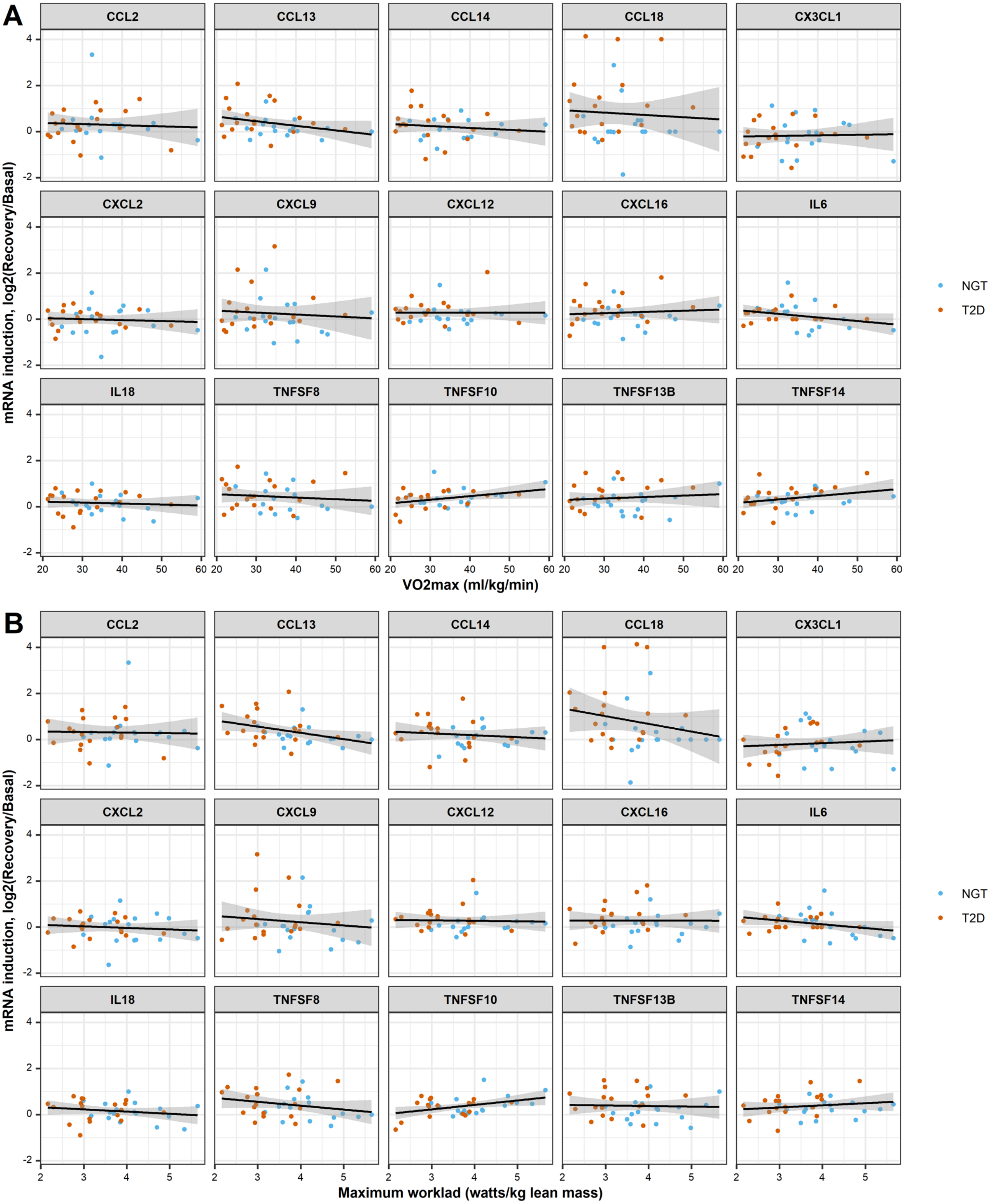

Fig, S1. Correlation between fitness level and cytokine induction. The fold-change of mRNA of cytokines (Recovery/Basal) was calculated and correlated to weight-normalized maximal oxygen uptake and maximal workload normalized to lean mass. Spearman correlation.

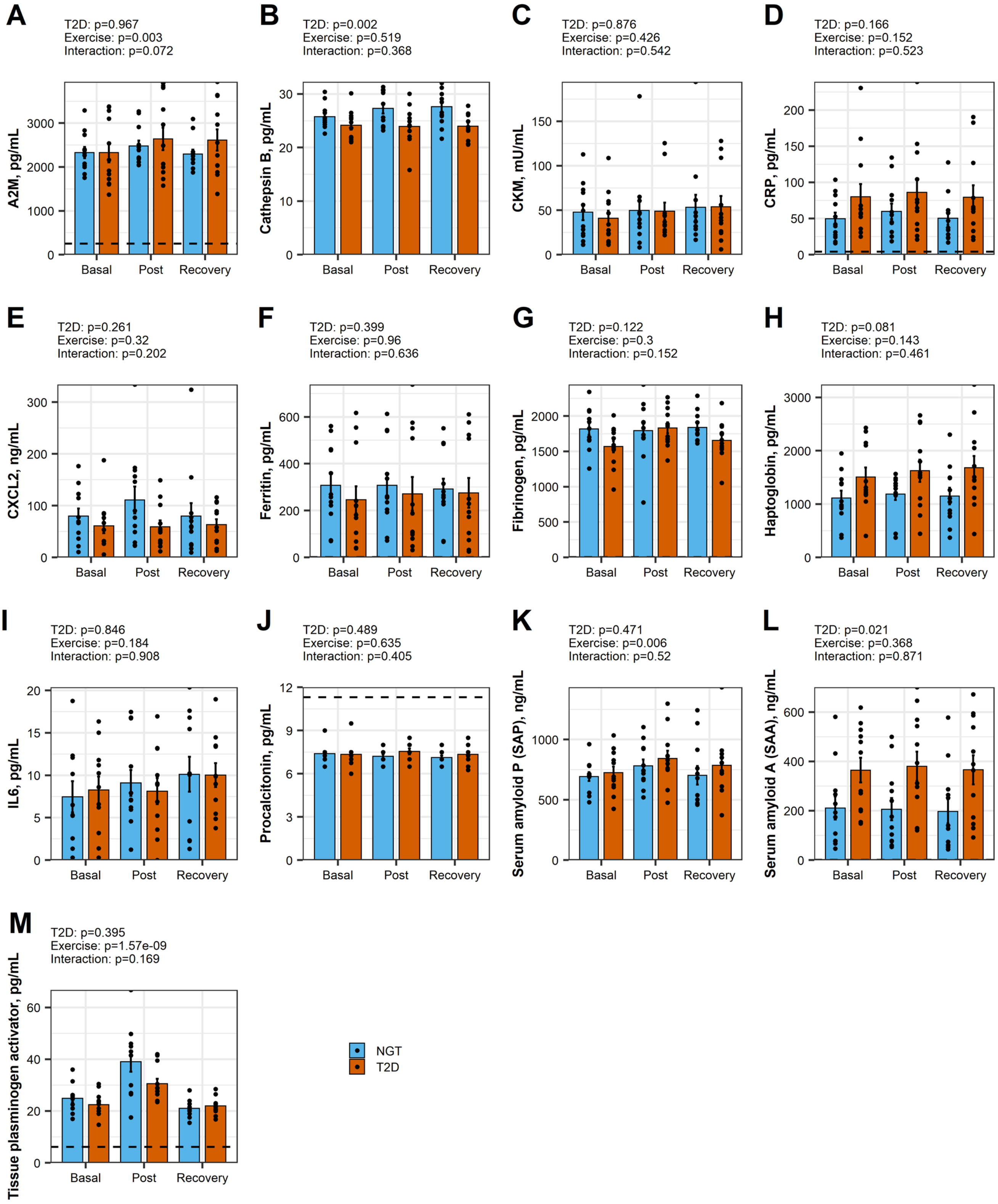

Fig, S2. Inflammatory markers and cytokine concentration in plasma. Cytokines were measured in plasma using immunoassays as described in methods. Data are mean ± SEM from individuals with normal glucose tolerance (NGT, n=9) or type 2 diabetes (T2D, n=10). Dashed lines indicate the lowest sensitivity of the assay.

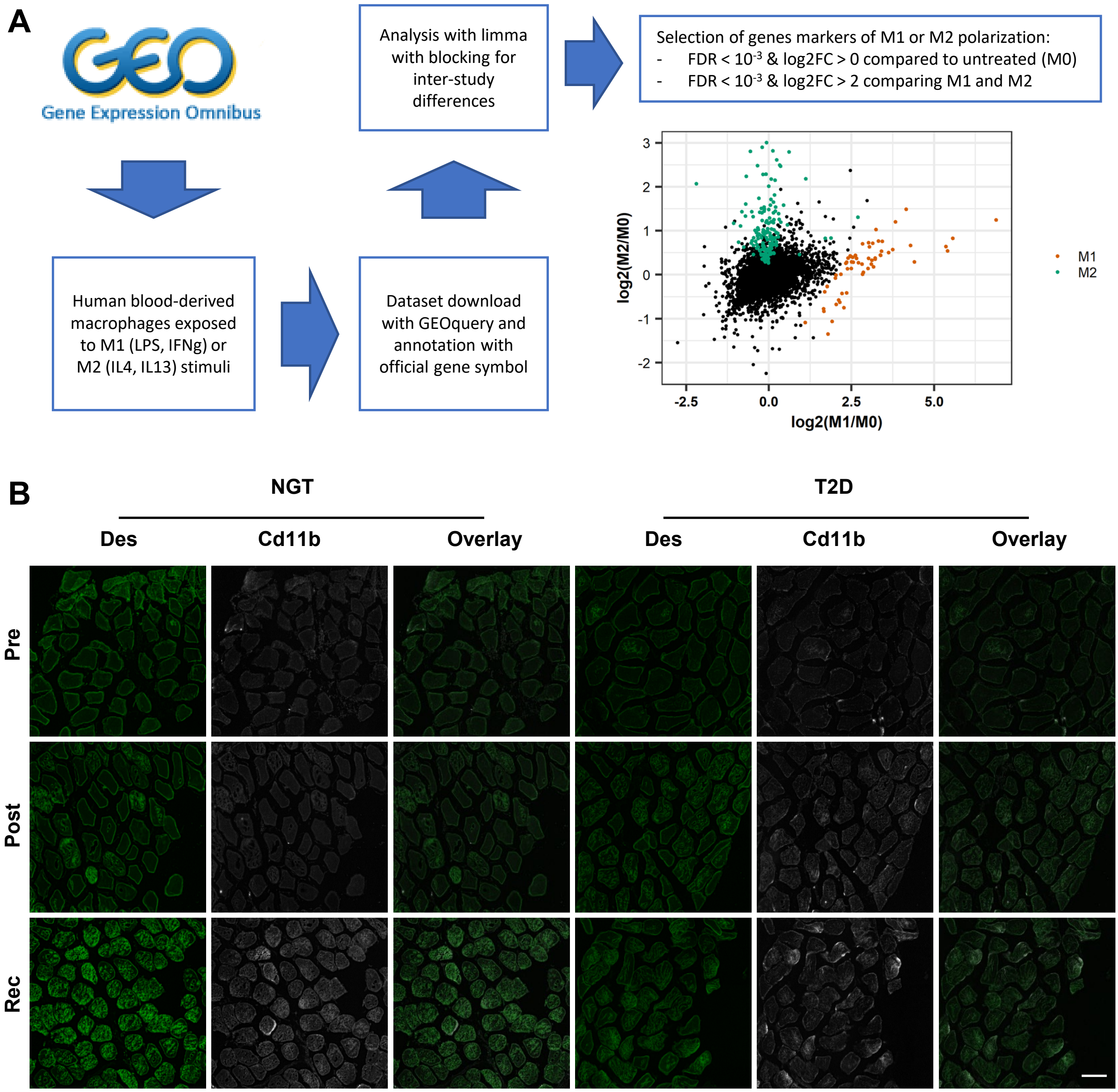

Fig. S3. Exercise-induced inflammation in individuals with type 2 diabetes is associated with immune cell polarization towards anti-inflammation. (A) Markers of M1 and M2 phenotypes were selected from publicly available data as described in methods. (B) Representative images of immunohistochemistry presenting myofibers labelled with desmin (Des, green) and immune cells labelled with Cd11b (white).

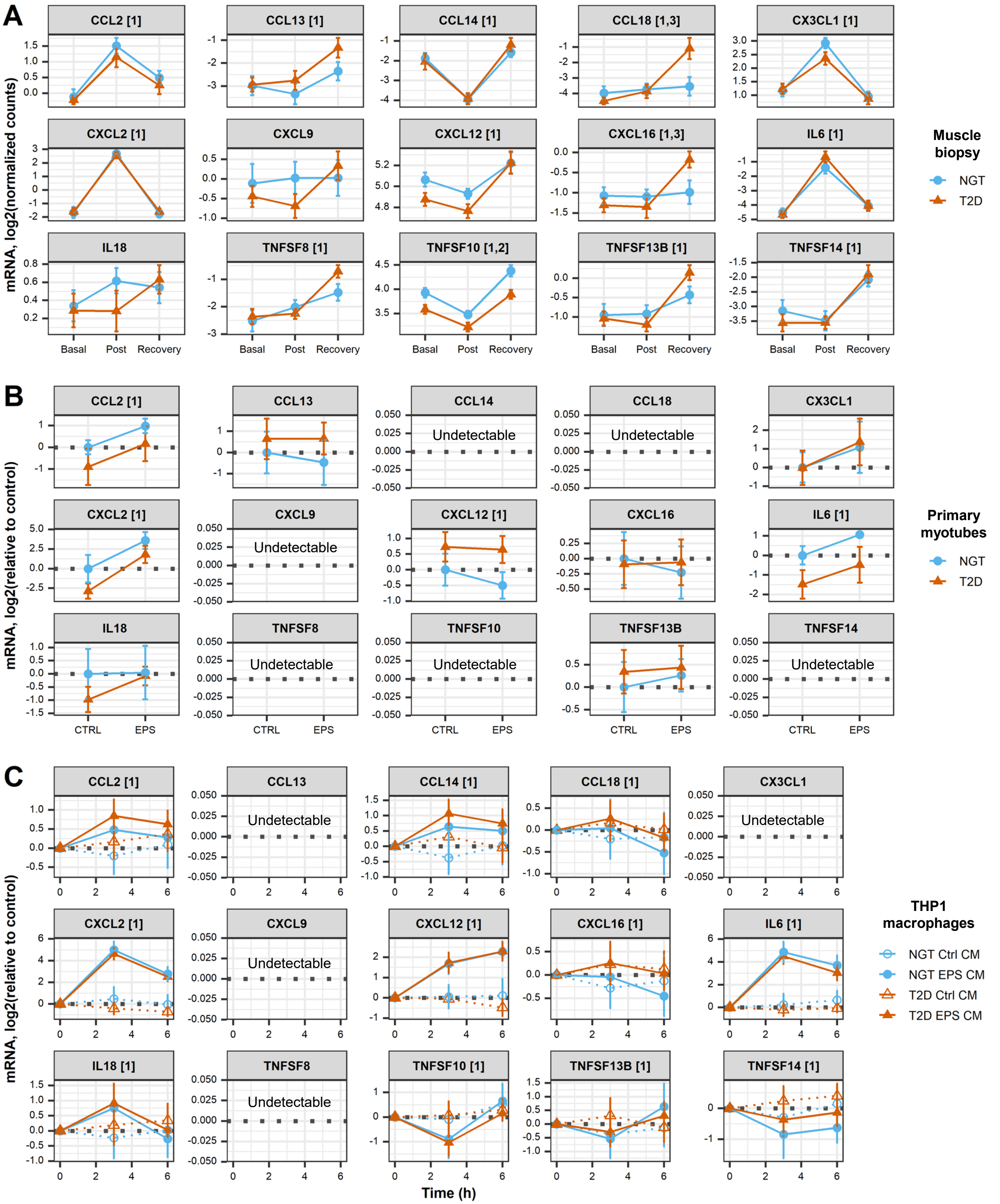

Fig. S4. Exercise-responsive cytokines in vivo and in vitro. (A) Cytokine mRNA response to acute exercise in skeletal muscle in vivo (see analysis on figure 2). Cytokines were selected based on a significant response to exercise (FDR<0.05) in either the normal glucose tolerant (NGT) or type 2 diabetic (T2D) group. (B) Cytokines mRNA response to electrical pulse stimulation (EPS, 40V, 3h) in primary human skeletal muscle cells. (C) Cytokines mRNA response in THP1 macrophages exposed to conditioned media from EPS-stimulated primary myotubes for 3 or 6h. Data is fold-change over control conditioned media.

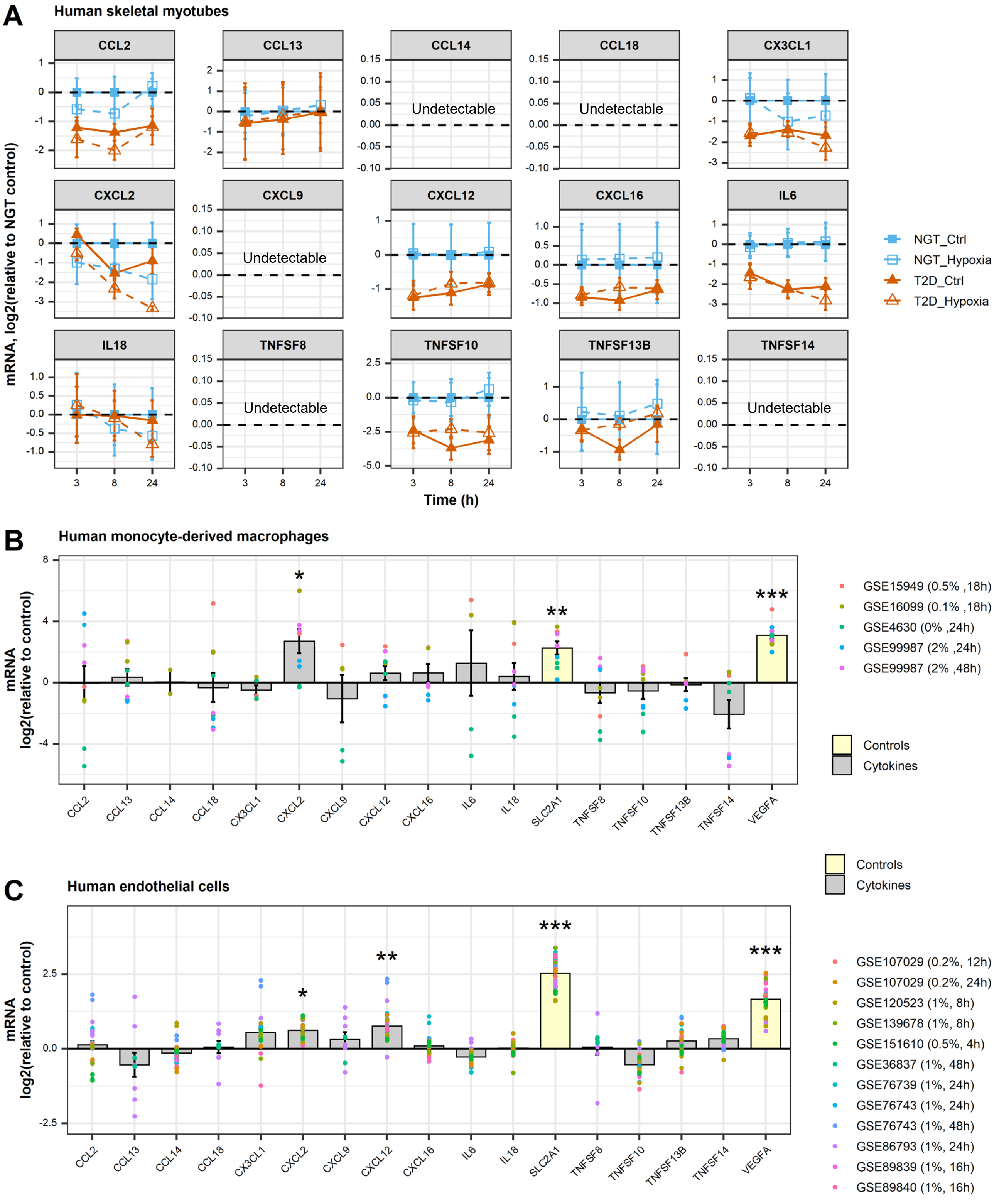

Fig. S5. Effect of hypoxia on cytokine expression in endothelial cells and macrophages in vitro. (A) Cytokines mRNA response to hypoxia (1% oxygen for 3, 8 or 24h) in primary human skeletal muscle cells. Quantitative PCR, myotubes derived from NGT (n=5) or T2D (n=6) donors. 2-way ANOVA on transformed data. [1] effect of EPS/Exercise (p<0.05). [2] effect of T2D (p<0.05), [3] interaction (p<0.1). (B-C) Publicly available data was downloaded and analyzed as described in methods for human monocyte-derived macrophages and endothelial cells. SLC2A1 and VEGFA were used as controls for the response to hypoxia. *FDR<0.05, **FDR<0.01, ***FDR<0.001.

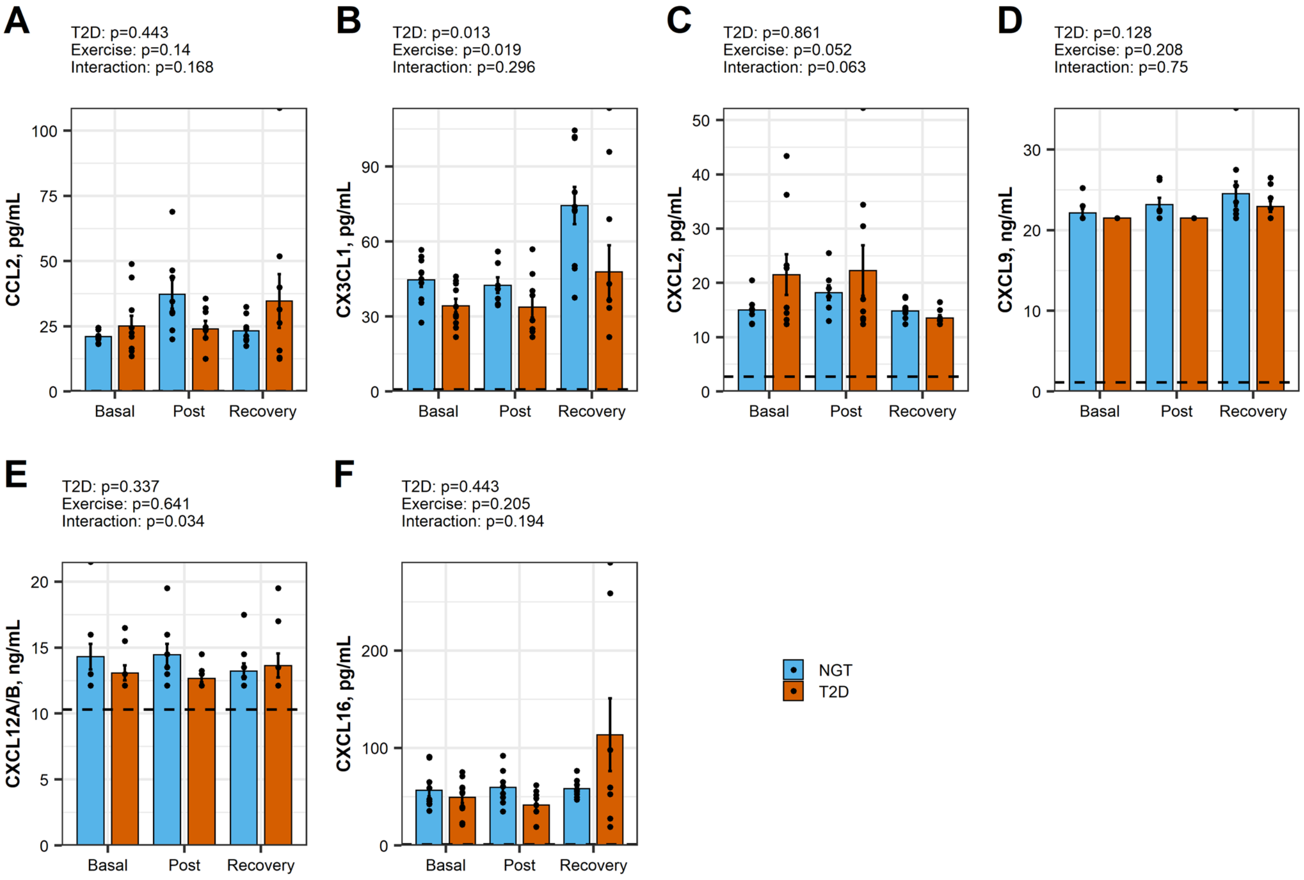

Fig, S6. Cytokine protein content in skeletal muscle. Cytokines were measured in protein extract from skeletal muscle biopsies as described in methods. Data are mean ± SEM from individuals with normal glucose tolerance (NGT, n=9) or type 2 diabetes (T2D, n=10). Dashed lines indicate the lowest sensitivity of the assay.
